# Microbial consortia for pesticide biodegradation: mechanisms, cross-class pathways, and translational challenges

**DOI:** 10.1007/s10532-026-10368-w

**Published:** 2026-09-14

**Authors:** Elango Lavanya, Johnson Iruthayasamy, Arunprakash Soodamani, Mareeswari Petchimuthu, Suganthi Angappan, Kavitha Murugavel, Karthikeyan Muthusamy

**Affiliations:** 1 Department of Plant Pathology, Tamil Nadu Agricultural University, Coimbatore, 641003 India; 2 Department of Plant Pathology, Agricultural College and Research Institute-Madurai, Tamil Nadu Agricultural University, Coimbatore, 641003 India; 3 Krishi Vigyan Kendra, Tamil Nadu Agricultural University, Ooty, The Nilgiris, 643001 India; 4 Department of Vegetable Science, Horticulture College and Research Institute, Tamil Nadu Agricultural University, Coimbatore, 641003 India; 5 Centre for Crop Health, University of Southern Queensland, Toowoomba, QLD 4350 Australia

**Keywords:** Microbial consortia, Pesticide biodegradation, Synergistic mineralization, Enzymatic degradation, Synthetic microbial communities, Environmental restoration

## Abstract

The use of synthetic pesticides estimated at 4.1 million metric tons annually worldwide, has led to widespread contamination of soils and aquatic environments, with documented risks to ecosystem integrity and human health. Physicochemical remediation methods are costly and often generate secondary pollutants and toxic intermediates, making microbial bioremediation a lower-residue alternative to physicochemical treatment. Because pesticide degradation in natural environments is rarely achieved by single microbial species, this review critically synthesizes evidence on multi-species microbial consortia—natural, synthetic, and genetically engineered for the biodegradation of six major pesticide classes: organophosphates, carbamates, pyrethroids, neonicotinoids, organochlorines, and triazines. Integration of the ecological mechanisms underlying consortium synergism (sequential metabolic cooperation, metabolic division of labour, biosurfactant-mediated bioavailability enhancement, horizontal gene transfer, and extracellular enzyme cooperation) with the enzymatic and genetic basis of degradation for each pesticide class, and evaluate how emerging tools like multi-omics profiling, CRISPR-based strain engineering, immobilisation technologies, synthetic consortium design, and AI-assisted optimisation are reshaping consortium design. Reported removal efficiencies are consistently higher for consortia than for monocultures across the studies reviewed here, though direct comparisons vary by pesticide class and experimental design. The review closes by evaluating the principal barriers to field-scale translation, strain persistence, ecological risk and regulatory approval, and monitoring of introduced strains and proposes a tiered framework for matching consortium design to contamination scenario. To date, this is the review to integrate ecological interaction mechanisms, class-specific enzymatic pathways, and translational technologies for pesticide-degrading consortia within a single framework.

**Graphical abstract:**

**Figure:**
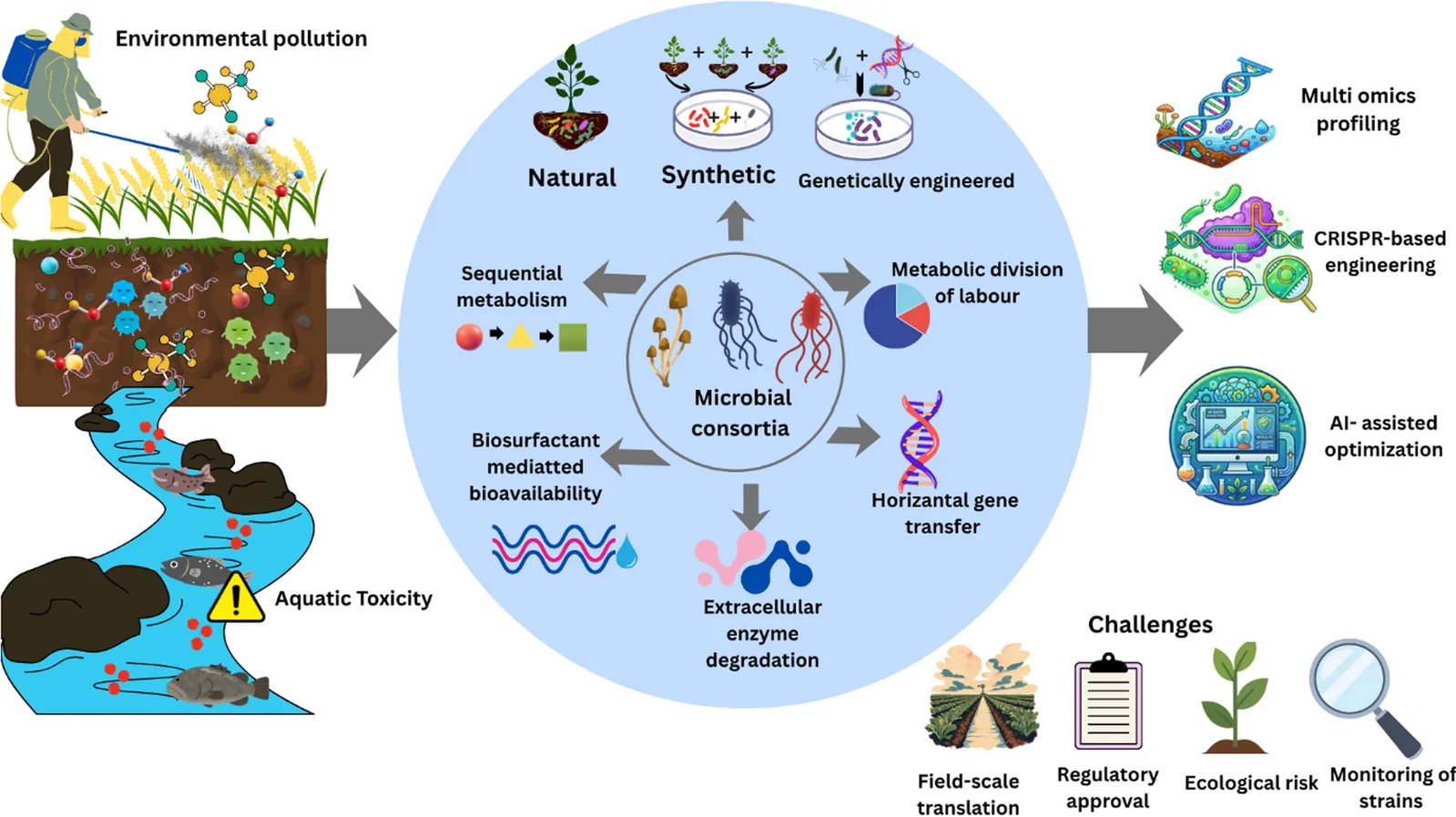

## Highlights

- Microbial consortia consistently outperform monocultures in pesticide removal.
- Enzyme and gene level pesticide class degradation pathways were reviewed.
- Synergistic interaction mechanisms responsible for consortium-based mineralization.
- New developments in CRISPR, AI, omics, and immobilization technologies in the field of consortium design were evaluated.
- New tiered system suggested for selection of consortia on a site-specific basis.

## Introduction

**Fig. 1:**
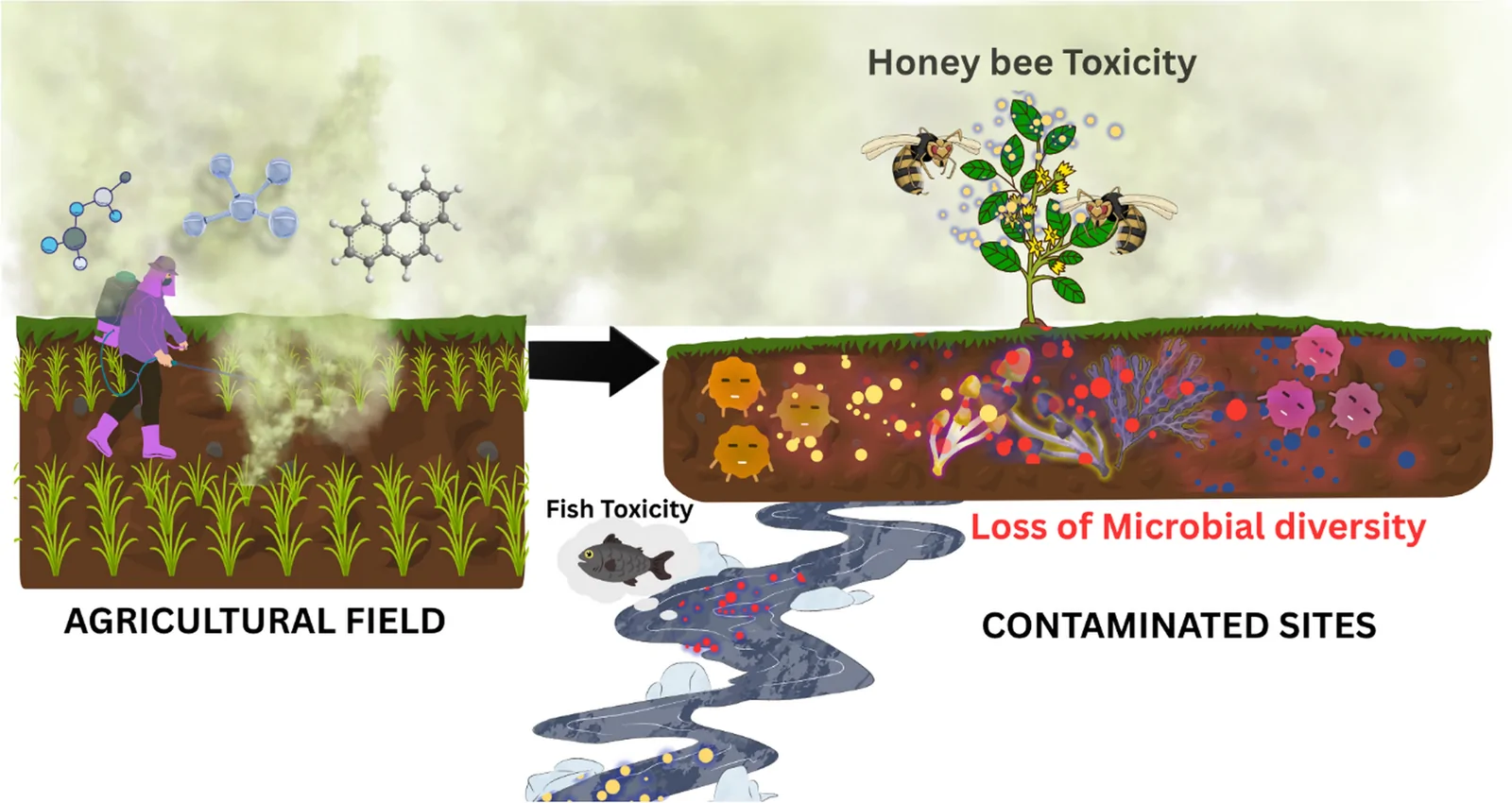
Pesticide residues move from soil and water into food chains, producing sublethal and lethal effects on non-target invertebrates, aquatic organisms, and vertebrates, with the highest documented sensitivity in pollinators and aquatic fauna

**Fig. 2:**
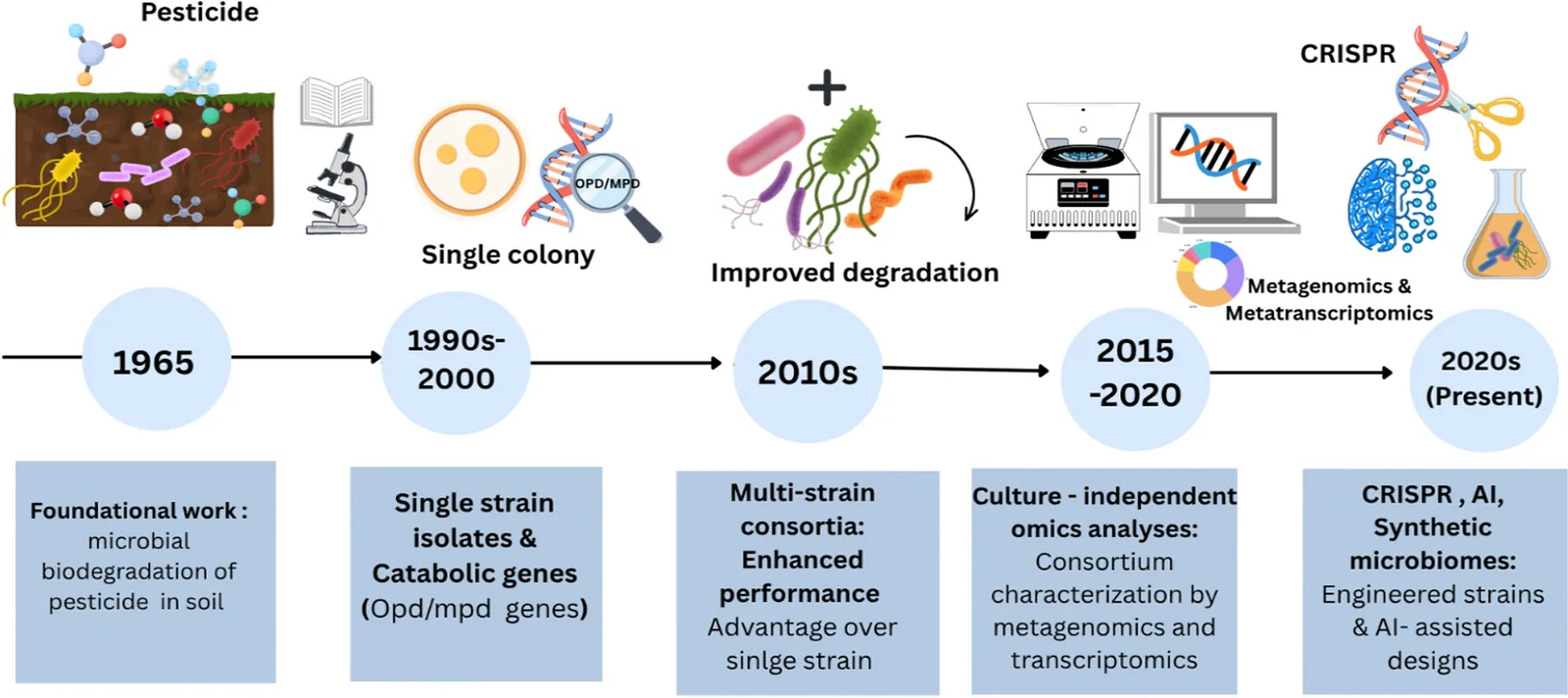
A chronological timeline of community-based bioremediation development of consortium-based bioremediation across ecological, biochemical and technological dimensions

Although synthetic pesticides are essential to modern agriculture, their worldwide use has placed an increasing burden on the environment, with chronic residues found in soils, groundwater, surface water, and food chains on every inhabited continent, and documented impacts on human health, ecosystem function and biodiversity, now removing more than 4.1 million metric tons from the environment each year (Tudi et al. 2021; Zhou et al. 2025). These residues exert a broad range of toxic effects on non-target organisms, disrupt trophic food webs, and pose a substantial risk of human exposure through diet and contaminated drinking water(Fig. 1) (Carvalho 2017).The traditional physicochemical remediation technologies are photodegradation, chemical oxidation, incineration, soil washing, which are either too costly or generate secondary contaminants and are unable to completely mineralize complex xenobiotic molecules (Ugrina and Juri 2023). However, microbial biodegradation takes advantage of the broad range of catabolic enzymes present across microbial taxa and breaks down the pesticide molecules to CO2, H2O, and inorganic ions, if the environmental conditions are favourable (Bhatt et al. 2021a, b). The use of microbial biodegradation for environmental applications, however, it has been greatly hindered by the limitations of single-strain systems. Although the individual microbial isolates are useful for mechanistic studies, they usually contain only part of a catabolic pathway, are not metabolically versatile to deal with mixed contamination and do not maintain competitive populations in complex soil environments. These constraints are not an accident, but are essential effects of the metabolic load resulting from the presence of the full complement of the catabolic pathways in a single organism (Lin 2022). This restriction is being overcome by microbial consortia, a cooperative multi-species community, whose combined enzymatic capacity allows complete mineralisation of pesticides which no single species could achieve. The catabolic potential of microbes has been exploited to clean up the environment since the pioneering work of Alexander (1965) and the interest in microbiological remediation of the environment has persisted since then (Fig. 2). One of the most basic lessons which have been learned over the decades of research is that the biodegradation of pesticides in natural ecosystems is hardly ever the monopoly of one single microbial species (Bose et al. 2021). Rather, complex pesticide structures are generally degraded by concerted metabolic activities of multi-species microbial consortia, where different community members complement each other with regards to enzyme capabilities (Mawad et al. 2023). Synergistic interactions of these consortia, which can be defined as interactions where the overall degradative performance is greater than the sum of individual contributions, are one of the crucial ecological principles that determine the fate of xenobiotics in the environment (French et al. 2020). Culture-independent molecular technologies have transformed how microbial consortia are characterised in xenobiotic degradation, revealing community composition and functional gene potential that culture-based isolation alone would miss; nevertheless, culture-dependent methods remain essential for defining the functional groups responsible for degradation, since gene presence detected by sequencing does not by itself confirm degradative activity**.** New degradation genes and metabolic pathways were largely undetectable by culture-based approaches, but have been discovered with more recent approaches to metagenomics, meta transcriptomics and single-cell genomics that have revealed the incredible complexity of communities that degrade pesticides (Jacoby et al. 2017). Although there is a large volume of literature related to microbial bioremediation, previous reviews have been either microbial strain specific on target pesticide classes, or general and overviews bioremediation approaches without mechanistic detail. Several recent reviews address components of this problem individually. Hassan and Ganai (2023) synthesized genome-editing and multi-omics approaches to pesticide bioremediation but do not treat the ecological basis of consortium synergism or provide site-selection guidance. Srivastava et al. (2026) organized recent advances by remediation strategy (bioaugmentation, bio stimulation, engineering) rather than by pesticide class or interaction mechanism. Reviews focused specifically on synthetic microbiome design (Sadvakasova et al. 2026) and on molecular/in-silico engineering (Shahid 2025) each cover a single technological strand without integrating the underlying ecological synergy mechanisms or cross-class enzymatic pathways (Ansar et al. 2026) address multi-omics approaches across emerging contaminants broadly, rather than pesticides specifically, and do not propose a consortium-type taxonomy or deployment framework. The present review differs from each of these by systematically mapping mechanistic synergy types to enzyme-level and gene-level degradation pathways across six major pesticide classes and by translating this evidence into a tiered Consortium Selection Framework that links site contamination severity to consortium type, assembly strategy, and realistic remediation period. “This review’s contribution is threefold: A four-tier framework linking contamination severity to consortium type and deployment strategy; Cross-consortium synergy pathways mapped at the enzyme and gene level across all six major pesticide classes rather than a single class; and An integrated technological system spanning multi-omics profiling, CRISPR-based engineering, immobilisation, synthetic consortium design, and AI-assisted optimisation, evaluated together rather than as separate strands.The review also highlights the main challenges and proposes an interdisciplinary path forward for the scaling-up and application of consortium-based bioremediation from the lab to the field for environmental restoration.

## Research methodology

**Fig. 3:**
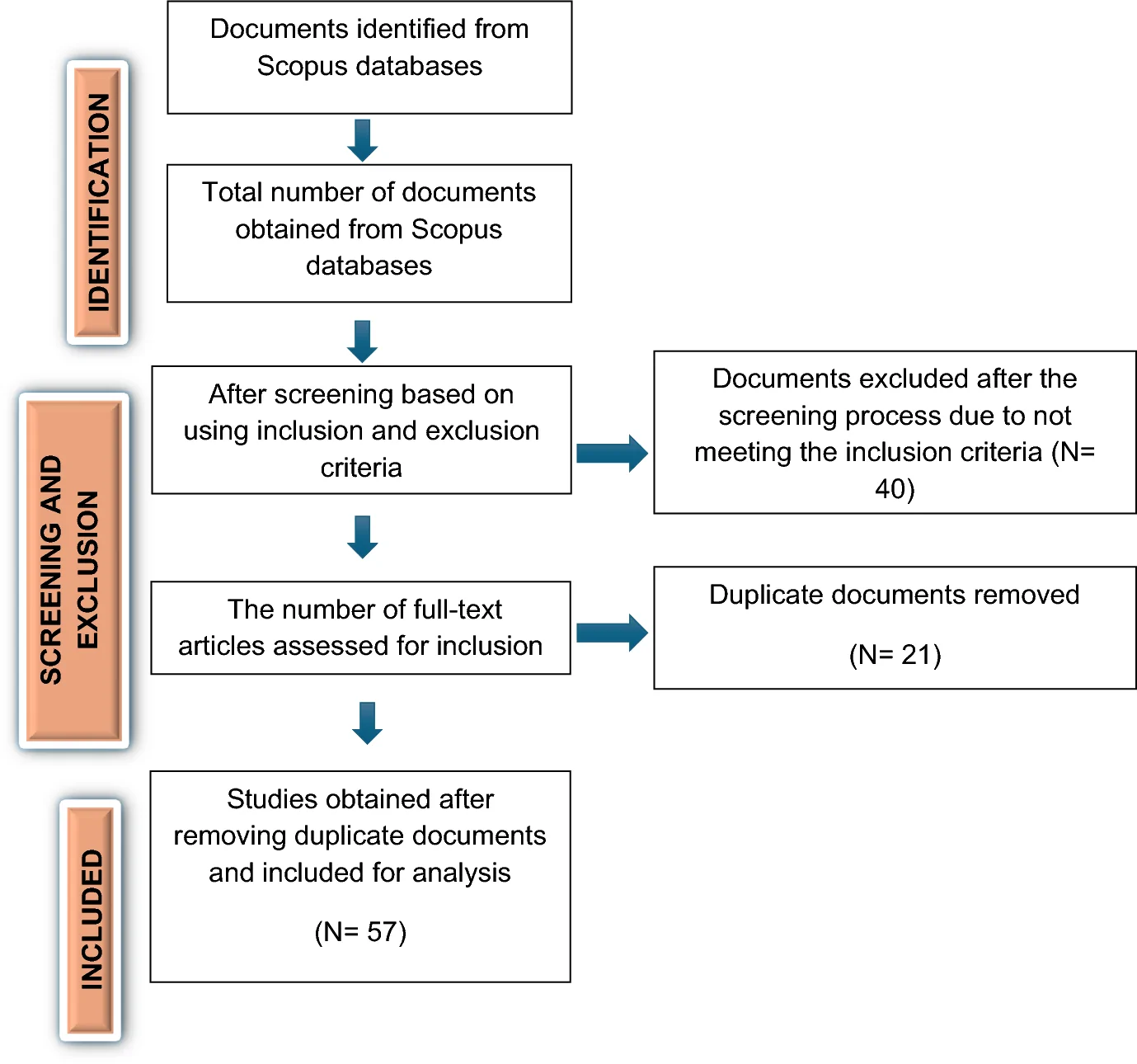
PRISMA table for literature selection process

The literature search and study selection were conducted using the Scopus database with the search strategy “pesticide degrading bacteria” AND “Microbial consortia” OR “Environment”. Scopus was selected as the sole database because of its comprehensive multidisciplinary coverage, rigorous indexing standards, and extensive collection of peer-reviewed literature across environmental, agricultural, and biological sciences, making it well suited for bibliometric analysis, Quantitative and qualitative review. The inclusion criteria comprised publications published between 2014 and 2026, written in English, available in the final publication stage, and indexed under the relevant subject areas, including Environmental Science, Agricultural and Biological Sciences, Biochemistry, Genetics and Molecular Biology, Chemistry, and Multidisciplinary. Eligible document types included research articles, review articles, and book chapters published in journals or books with open access. Publications outside these criteria, including short surveys, non-English documents, book series, articles in press, restricted-access publications, and studies belonging to unrelated subject areas such as Engineering, Medicine, Computer Science, Veterinary Sciences, Physics, Mathematics, Health Professions, Pharmacology, Nursing, Toxicology, Earth and Planetary Sciences, Energy, Decision Sciences, and Chemical Engineering, were excluded (Table 1) for bibliometric and qualitative analysis. The complete literature selection process is illustrated in Fig. 3. This strategy ensured the systematic identification and selection of relevant studies, providing an evidence-based foundation for the subsequent analysis and discussion.

**Table 1:** The inclusion and exclusion criteria for literature search strategy

Criteria	Inclusion	Exclusion
Time period	2014–2026	< 2014
Subject area	Environmental science, agricultural and biological science, biochemistry, genetics and molecular biology, chemistry, multidisciplinary	Immunology and microbiology, earth & planetary science, engineering, energy, medicine, computer science, decision sciences, chemical engineering, nursing, toxicology & pharmaceutics, pharmacology, health professions, mathematics, veterinary, physics & astronomy
Document type	Article, review, book chapter	Short survey
Languages	English	Non-english
Source type	Journal, book	Book series
Publication stage	Final	Press
Open access	All open access	Green, gold, hybrid gold
Screening		
Title and abstract	Existence of predefined keywords in the title, abstract, or keywords part of the paper	
Full text	Included relevant papers that discuss the predefined keywords

## Why consortia outperform single strains: evidences and mechanisms

The basic benefits of microbial consortia over single-strain systems can be best understood by comparing performance directly across functional parameters. These differences are summarised in Table 2, based on evidence gathered from the studies examined in this review.

**Table 2:** Comparative table for benefits of microbial consortia over single-strains

Pesticide	Consortium	Single-strain	Toxic-intermediate accumulation	Environmental adaptivity	References
Chlorpyrifos	65.9% in 20 days (four-member consortium, 500 mg L⁻^1^)	< 50% by best single strain, same conditions	TCP formed in the soil phase	Consortium maintained activity across liquid-culture and soil-phase conditions at pH 7, 37 °C	Sasikala et al. (2012)
Chlorpyrifos	91% in 6 days (four-strain Bacillus consortium, 100 ppm)	Not tested against matched single-strain control	Not reported	Not reported	Varghese et al. (2021)
Imidacloprid	82% (MSM); 75.7% (soil slurry); 69% (soil)—three-strain consortium	64%, 68%, 62% respectively, *Pseudomonas* sp. alone	Desnitro-imidacloprid -intermediate for this pesticide	Consortium tested across three matrices -MSM, soil slurry, soil	Negi et al. (2014)
Diflufenican	74.4% (four-member synthetic consortium, liquid medium)	70.1% by best single strain (D1)	Not reported	Four-member synthetic consortium tested in liquid medium only—no field/soil adaptation data reported	Książek-Trela et al. (2025)
Pyrene (model xenobiotic)	98% removal, five-member mangrove consortium	< 60% by individual strains	No toxic intermediate	adapted to a saline/tidal environment	Ahmad et al. (2023)
Glyphosate	100% in 36 h, consortium YS622 (50 mg L⁻^1^)	Not reported with matched single-strain control	AMPA is the known glyphosate intermediate	Consortium YS622 achieved complete degradation in 36 h at 50 mg/L—fast-acting but adaptation range untested at other concentrations	Chen et al. (2025)

These comparative advantages collectively explain why naturally enriched and synthetically designed microbial consortia have become the preferred approach in contemporary bioremediation research and represent the logical direction for scale-up bioremediation applications (Bhatt et al. 2021a, b; Liu et al 2023). The underlying reason is ecological rather than incidental: in microbial consortia, synergistic interactions among microorganisms and other soil biota- both microfauna and mesofauna were sustain the biochemical activities that single strains cannot maintain alone, serving as major agents of nutrient conversion and energy exchange across diverse environmental conditions. Soil bacterial communities have been shown to tolerate pesticide exposure and adapt metabolically to pesticide-contaminated soil over time (Ahmad et al. 2024; Srivastava et al. 2026). This adaptive capacity operates through metabolic division of labour: by distributing the steps of a degradation pathway across several community members, no single organism bears the full metabolic burden of complete pathway operation (Li et al. 2024a, b). Each consortium member typically expresses only a specialised subset of catabolic enzymes, with the balance of catabolic investment and metabolic return sustained through cross-feeding interactions among partners (Alidoosti et al. 2024).

## Types of microbial consortia

**Fig. 4:**
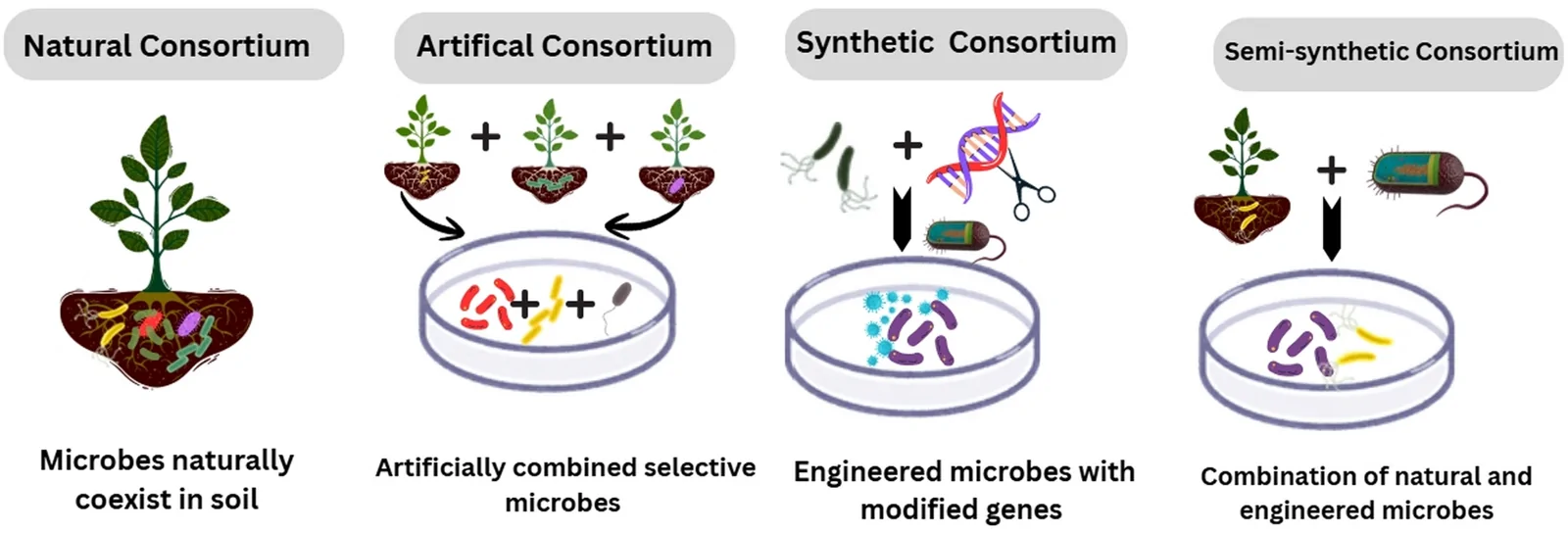
Microbial consortia used in pesticide bioremediation fall into three assembly types- natural, synthetic, and semi-synthetic distinguished by their origin and the degree of deliberate strain selection, which in turn determines their catabolic breadth and field deploy ability

Based on their origin, composition and intentional assembly, microbial consortia are broadly classified into four major groups: Naturally enriched consortia, Artificial consortia, Synthetic consortia, Semi-synthetic consortia (Table 3) (Fig. 4). Ecosystems, when exposed to a long-term pesticide exposure, will tend to become enriched naturally, with the enrichment of the community resulting from the selective pressure exerted by the pesticides and favouring the degradation-competent species. Such consortia have the ecological benefit that they are pre-adapted for the soil chemistry and indigenous competitive conditions. Artificial consortia, on the other hand, are made up of strains with complementary functional properties that are intentionally combined, and can include organisms that do not co-occur naturally within a single ecosystem, as in a microcosm (Massot et al. 2022). The most advanced category are synthetic microbial consortia, which consist of two or more genetically engineered strains that are designed to carry out a specific metabolic goal while communicating with each other in a controlled manner and dividing up the metabolic tasks (McCarty and Ledesma-Amaro 2019). Semi-synthetic consortia are collections of naturally occurring indigenous strains with synthetic engineered strains that have the advantages of both ecological fitness as well as enhanced metabolic capacity. These categories are not mutually exclusive as many applied bioremediation systems adopt a hybrid approach of enriching the naturally occurring community with specialist strains rationally selected to solve specific bottlenecks in degradation (Padmaperuma et al. 2020; Ahmad et al. 2024). The relationship between the microbial consortia can either be cooperative or non-cooperative. Positive relationships include those of co-operative interactions such as mutualism, commensalism, and symbiosis that involve one or both microbial members that benefit and result in an overall increase in the functioning of the community. Non-cooperative relationships like competition, predation, ammensalism and parasitism can cause a decrease in the number of individuals belonging to the consortia, on the other hand (Zapalski 2011). The balance of this type of interaction between the microbes could ultimately depend upon how the consortia form regarding chemical stressors like the presence of pesticides.

**Table 3:** The different categories of microbial consortia based on their origin and assembly adapted from (Massot et al. 2022) and (Padmaperuma et al. 2020)

Type of microbial consortia	Description	Key characteristics	Applications
Natural microbial consortia	Microbial communities that naturally coexist and interact within a shared environment	Ecologically stable, adapted to local conditions, high functional diversity	Soil nutrient cycling, natural pesticide degradation, ecosystem sustainability
Artificial microbial consortia	Deliberately assembled consortia composed of selected microbial strains that may not naturally coexist	Designed for specific functions, controlled composition, complementary metabolism	Targeted pesticide degradation, bioremediation, agricultural soil improvement
Synthetic microbial consortia	composed of two or more genetically modified microorganisms engineered for specific tasks	High functional precision, enhanced metabolic pathways, controllable performance	Advanced biodegradation, metabolic engineering, environmental biotechnology
Semi-synthetic microbial consortia	combining naturally occurring microorganisms with genetically modified strains	Balance of ecological adaptability and engineered efficiency	Agricultural applications, soil remediation, stress-resilient biodegradation systems

## Pesticide-degrading bacteria and fungi: diversity and enzymatic potential

Many genera of bacteria commonly present in soil have been reported to have pesticide degrading potential such *Pseudomonas, Achromobacter, Arthrobacter, Bacillus, Alcaligenes, Burkholderia, Klebsiella, Stenotrophomonas* (Lescano et al. 2022), *KosaKonia, and Enterobacter* (Li et al. 2024a, b) (Wang et al. 2022) (Table 3). These are responsible for the breakdown of pesticides in several ways through different enzyme systems and adaptive metabolism. (Table 4). The species of fungi also play a significant role in the pesticide bioremediation as they can synthesize high levels of extracellular and intracellular enzyme. A few mushrooms fungi are known to be stable and degrade several classes of pesticide such as phenylamides, triazines, phenyl urease, dicarboximides, organophosphates, organochlorines, and carbamates: Mushroom fungi are *Agrocybe semiobicularis, Auricularia auricula, Coriolus versicolor, Dichomitus squalens, Flammulina velutipes, Hypholoa fasciculare, Pleurotus ostreatus, Stererum hirsutum, and Antrodiella discolor.* The ability of white rot fungi to degrade recalcitrant pesticides like DDT, Lindane, Endosulfan, Aldrin, dieldrin, atrazine and γ-hexachlorocyclohexane is noteworthy. The presence of a large body of enzymes in the enzymatic machinery of the white-rot fungi is responsible for the diversity of white-rot fungi that produces extracellular ligninolytic enzymes (lignin peroxidase, manganese peroxidase, versatile peroxidase, and laccase) and intracellular system (cytochrome P450 monooxygenases). Fungi including *Trametes versicolor* are known to break down water soluble and hydrophobic pesticides through a coordinated extra and intracellular mechanism and hence play an important role in bioremediation of complex pesticide contaminated environment (Chakraborty et al. 2025).

**Table 4:** Reported pesticide degradation by microbial consortia, highlighting their consortium composition, pesticide and experimental matrix, degradation time and efficiency, detected metabolites, analytical methods

Microbial consortium	Pesticide degraded	Matrix/scale	Time	Degradation efficiency	Metabolites measured	Analytical method	References
*Pseudomonas* sp., *Acinetobacter* sp., *Bacillus* sp., *Stenotrophomonas* sp. (bio-mixture-derived consortium)	Atrazine	Liquid culture medium, lab scale	Not reported	≥ 90% degradation	Not reported	Not reported	(Góngora-Echeverría et al. 2020)
*Pseudomonas* sp., *Acinetobacter* sp., *Bacillus* sp., *Stenotrophomonas* sp. (bio-mixture-derived consortium)	Carbofuran	Liquid culture medium, lab scale	Not reported	≥ 90% degradation	Not reported	Not reported	(Góngora-Echeverría et al. 2020)
*Pseudomonas* sp., *Acinetobacter* sp., *Bacillus* sp., *Stenotrophomonas* sp. (bio-mixture-derived consortium)	Glyphosate	Liquid culture medium, lab scale	Not reported	≥ 90% degradation	Not reported	Not reported	(Góngora-Echeverría et al. 2020)
*Pseudomonas* sp., *Enterobacter* sp., *Bacillus* sp. (enriched consortium YS622)	Glyphosate (50 mg L⁻^1^)	Liquid culture medium, lab scale	36 h	Complete (100%) degradation	AMPA	Not reported	(Chen et al. 2025)
*Pseudomonas*, *Ochrobactrum*, *Comamonas* spp. (glyphosate-degrading enriched consortium)	Glyphosate (400 mg L⁻^1^)	Liquid culture medium, lab scale	7 days	46.4–100% degradation	AMPA	Not reported	(Chen et al. 2025)
*Sphingomonas* sp., *Pseudomonas* sp., *Rhodococcus* sp. (consortium TA01)	Difenoconazole (50 mg L⁻^1^)	Liquid culture medium, lab scale	5 days	83.87% degradation	Not reported	Not reported	(Zhang et al. 2025)
*Pseudomonas* sp., *Arthrobacter* sp., *Paenarthrobacter* sp., *Bacillus* sp. (consortium AT1)	Acetochlor	Not reported	12 days	Nearly complete (~ 100%) degradation	Not reported	Not reported	(Dai et al. 2025)
*Azospirillum* sp., *Ochrobactrum* sp., *Sphingobium* sp., *Sphingomonas* sp. (paddy soil-derived consortium)	Fipronil	Soil microcosm	Not reported	> 80% degradation	Not reported	Not reported	(Li et al. 2024a, b)
*Azospirillum* sp., *Ochrobactrum* sp., *Sphingobium* sp., *Sphingomonas* sp. (paddy soil-derived consortium)	Thiobencarb	Soil microcosm	Not reported	> 85% degradation	Not reported	Not reported	(Li et al. 2024a, b)

These examples demonstrate that consortia can deal with mixtures, function over an environmental gradient and frequently. These exhibit higher rate and extent of degradation than individual strains Góngora-Echeverría et al. 2020).

## Mechanisms of pesticide biodegradation by microbial consortia

The overall metabolic capability of microbial consortia is an important factor in breakdown of pesticide as these can easily convert complex compound of pesticide into easily degradable form. In the natural environment pesticide is frequently encountered as individual substrates, but will be encountered in combination with a range of different microbial communities in which the degradation process can be a series of coordinated and subsequent biochemical reactions. The biodegradation processes based on consortium approach are based on this kind of cooperation metabolism. (Massot et al. 2022) (Bhatt et al. 2021a, b).

The mechanistic principles are manifested differently within pesticide chemical classes because of the diversity of target molecules in nature, and their application to the degradation of each major pesticide family is discussed below.

### Toxicity of degradation intermediates and the role of consortia in complete detoxification

**Fig. 5:**
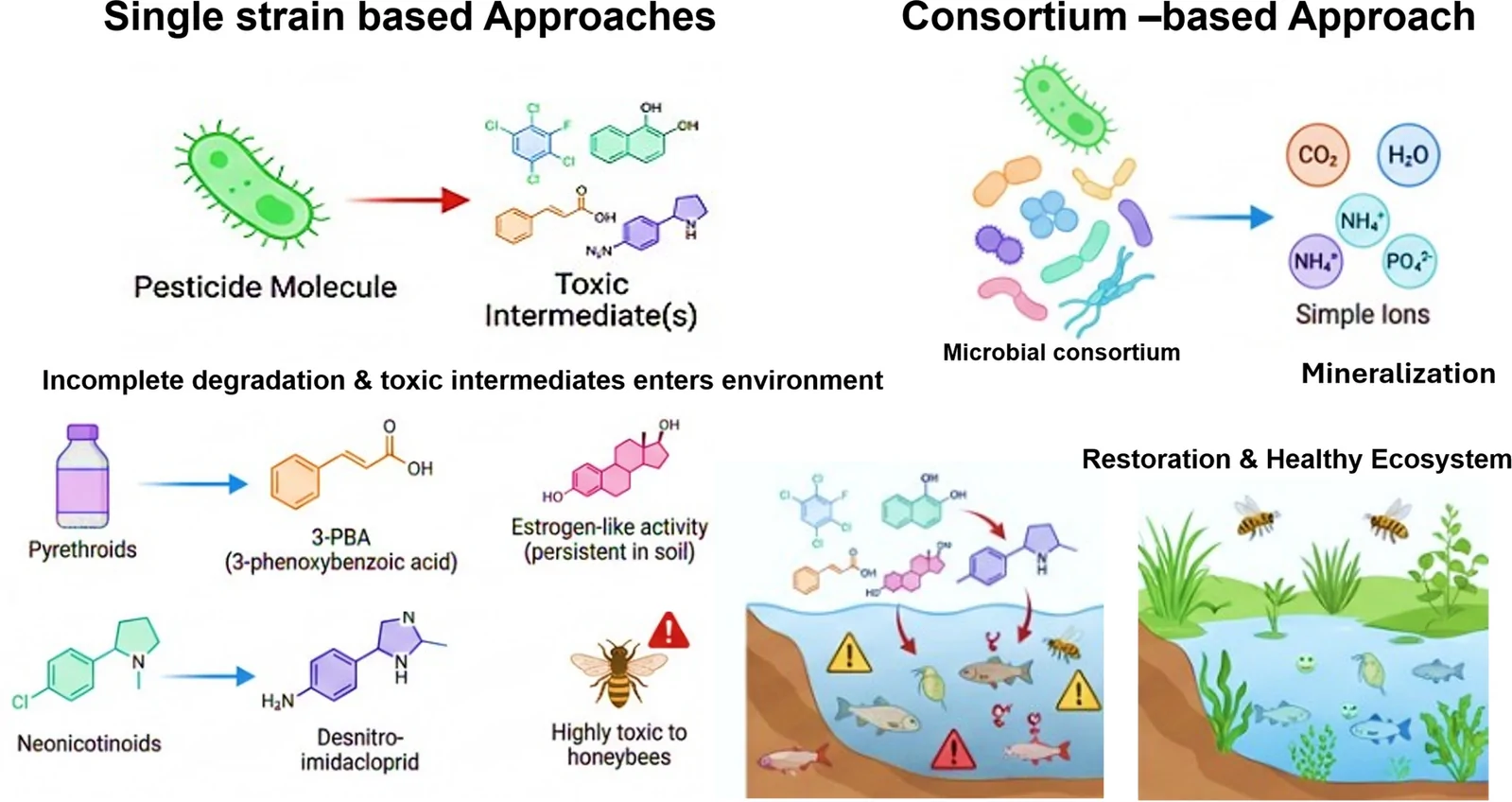
Single-strain degradation may result in the accumulation of persistent or toxic intermediates, whereas metabolically complementary microbial consortia can promote further degradation and mineralization into simpler, less harmful products, thereby reducing ecological risks and supporting ecosystem restoration

One aspect of pesticide bioremediation that is often overlooked is the ecotoxicological characteristics of degradation products as compared to the parent compound. Single-strain systems are often not as effective in completely degrading the pesticide, causing the generation of transformation products that can be as toxic or more toxic than the original pesticide. The main hydrolysis product of chlorpyrifos is 3,5,6-Trichloro-2-pyridinol (TCP), which is known to have antimicrobial activities at concentrations higher than 10 mg/L and moderate aquatic toxicity (LC_50_ Daphnia magna: 0.36 mg/L), which may outweigh the ecotoxicological effects of the parent compound in aquatic microcosms (Huang et al. 2021). Likewise, 1-naphthol from the hydrolysis of carbaryl is a known aquatic toxicant and endocrine disruptor and 3-phenoxy benzoic acid (3-PBA) from the metabolism of pyrethroids has oestrogen-like activity which is long-lasting in soil (Kaur and Patowary 2026). It is suggested that the bioremediation of neonicotinoid contaminated soils by single degraders may lead to an increase in ecological damage because of the high toxicity of the major neonicotinoid metabolites, such as desnitro-imidacloprid to honeybees, compared to the parent molecule itself (DPR 2024). (Figure 5) All of these results agree that the standard of good bioremediation should be set beyond just the removal of the parent compound to complete mineralization or proven detoxification of all transformation products. The use of microbial consortiums to reduce the build-up of pathway intermediates and allow for complete mineralization of the substrate is a key benefit to ensure that the intermediate metabolites are consumed instead of leaving the system to contaminate the environment, and thus provide true ecological restoration, not contaminant substitution (Xu et al. 2020; Li et al. 2022) (Table 5).

**Table 5:** Known transformation products of major pesticide classes, their reported toxicological relevance, and consortium-level detoxification

Parent pesticide	Key transformation product(s)	Reported environmental/toxicological relevance	Consortium-level detoxification confirmed?
Chlorpyrifos	TCP (3,5,6-trichloro-2-pyridinol)	Antimicrobial above 10 mg/L; moderate aquatic toxicity, Daphnia magna LC50 0.36 mg/L	Yes, where consortium includes TCP-degrading not confirmed for single strains
Carbaryl	1-Naphthol	Aquatic toxicant; reported endocrine-disrupting activity	Yes, consortium BP16 (95% mineralization, 96 h); not confirmed as general rule
Pyrethroids (cypermethrin etc.)	3-Phenoxybenzoic acid (3-PBA)	Oestrogenic activity reported; persistent in soil relative to parent compound	Partially -consortium YC-L1 degrades 3-PBA, but many pyrethroid-hydrolysing strains cannot
Glyphosate	AMPA (aminomethylphosphonic acid)	More persistent than glyphosate; frequently detected in soil/water; IARC flagged for review alongside glyphosate	Not systematically confirmed in consortium studies reviewed here which is a research gap
Imidacloprid/acetamiprid	Desnitro-imidacloprid; 6-chloronicotinic acid	Desnitro-imidacloprid more toxic to honeybees than parent compound (DPR, 2024)	Not systematically confirmed; single-strain bioremediation flagged as a possible net ecological harm (DPR, 2024)

Known transformation products of major pesticide classes with reported ecotoxicological relevance, and whether consortium-level detoxification (rather than mere parent-compound disappearance) has been directly confirmed in the literature reviewed. Where confirmation is absent, this is stated as a gap rather than assumed.

### Synergistic interactions in microbial communities

**Fig. 6:**
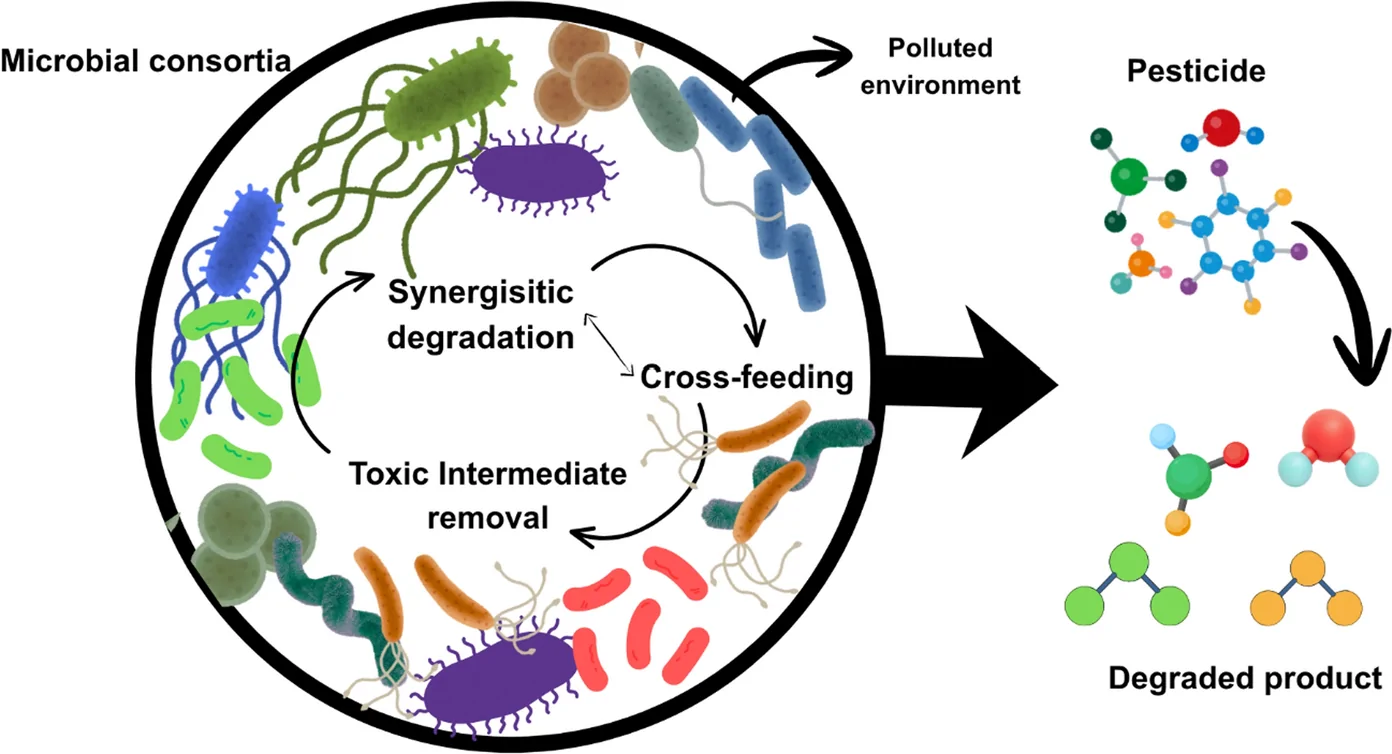
The six synergistic mechanisms by which microbial consortia outperform monocultures—sequential metabolic cooperation, biosurfactant-mediated bioavailability enhancement, competitive exclusion/niche partitioning, self-regulation, metabolite cross-feeding, and extracellular enzyme cooperation which operating alone or in combination to achieve complete pesticide mineralisation

Microbial consortia are made up of different species of microbes that cooperate with each other in ways that yield results that are greater than the sum of the parts, this is known as synergism. In relation to pesticide degradation, synergism is the increases in degradation rates, degradation of a wider range of substrates, reducing environmental stress, and better mineralization than monocultures (Massot et al. 2022). Functional consortia synergistically degrade and there are six key mechanistic pathways that may act alone or in combination (Fig. 6).

### Sequential metabolic cooperation

The metabolic complementarity of pesticide-degrading consortia is the most basic reason for synergy, namely the members of the consortium have different enzymatic activities that are complementary and together results in complete degradation of the structure of the pesticide. Pesticides are generally very complex molecules with more than one functional group and ring structure, and are not totally degraded by any single microbial species. Sequential synergism in the degradation of an organochlorine pesticide (1,1,1-trichloro-2,2-bis(4-chlorophenyl) ethane (DDT)) has been documented in a consortium consisting of different strains (Tang et al. 2021). DTX bacteria, which can release dehalogenases, reductively dechlorinate DDT to DDD (1,1-Dichloro-2,2-Bis(4-chlorophenyl) ethane) or oxidatively convert DDT to DDE (1,1-Dichloro-2,2-Bis(4-chlorophenyl) ethylene) under aerobic conditions. These primary metabolites are then mineralized by bacteria that are unable to act directly on the parent DDT molecule, but can degrade aromatic compounds. In consortia for DDT degradation, anaerobic reductive dechlorination bacteria and aerobic ring-cleaving bacteria have been demonstrated to be much more effective in DDT degradation than the anaerobic reductive dechlorination bacteria alone (Bhatt et al. 2022). The microbial consortia often also contain organophosphate hydrolase (OPH)-producing bacteria which typically break chlorpyrifos down into first metabolites, 3,5,6-trichloro-2-pyridinol (TCP) and diethyl thiophosphate (DETP). This potentially toxic intermediate, TCP, is further broken down by members of the consortium that contain enzymes to degrade chlorophenols, such as phenol hydroxylase, catechol dioxygenase and chlorocatechol dioxygenase. The cooperative degraders can remove TCP from the system, which prevents its accumulation to inhibitory concentrations, and thermodynamically pushes the forward reaction, thus maximizing the overall rate of chlorpyrifos transformation (Li et al. 2023). This mechanism is characterised using degradation pathway steps by the different consortium members with intermediates produced by one member as substrates for another consortium member. Sequential cooperation prevents the build-up of potentially toxic or inhibitory intermediates and allows the entire pathway to be covered even if none of the organisms contains all enzymes. For instance, in the case of pyrene degradation, the five-member consortium from mangroves of Thailand exhibited sequential cooperation in which *Mycobacterium* sp. was involved in the hydroxylation of pyrene to produce intermediates, *Novosphingobium pentaromativorans* in the removal of phthalate, and *Ochrobactrum* sp. in the removal of protocatechuate. This metabolic relay system resulted in 98% removal of pyrene in comparison to the individual strains which achieved < 60% removal (Ahmad et al. 2023). The sequential-cooperation mechanism is among the most rigorously demonstrated in this review, being confirmed by direct metabolite tracking (LC–MS/GC–MS identification of pathway intermediates) and, in several cases, by gene-level characterization of the responsible enzymes (e.g., linA/linB dehalogenases, opd/mpd hydrolases). However, almost all supporting studies were conducted in liquid or mineral salts medium over days to weeks; soil-matrix or field-scale confirmation of the same sequential pathways remains comparatively rare, and sorption of pathway intermediates to soil organic matter may alter the cross-feeding kinetics observed in culture.

### Biosurfactant production and bioavailability enhancement

Hydrophobic pesticides with low water solubility (pyrethroids and polycyclic aromatic hydrocarbons) have a low bioavailability for degrading microorganisms. Some members of the consortium are experts in the production of biosurfactants, which are amphiphilic molecules that will dissolve hydrophobic substances and thus make them more available to other degraders (Zhu et al. 2025). *Bacillus* sp. A consortium of microorganisms that degrade pyrene, called pyrene degraders, can produce biosurfactants with a high degradation efficiency that enhance the solubility and bioavailability of pyrene, which boost the uptake of pyrene by the degraders (Qutob et al. 2022). Likewise, there have been consortia developed for the degradation of hydrophobic organochlorines and pyrethroids that include rhamnolipid-producing *Pseudomonas*, and addition of the biosurfactant to the consortia has resulted in a 40–60% higher degradation rate than has been achieved in surfactant-free consortia (Zhu et al. 2025).

### Competitive exclusion & niche partitioning

Niche partitioning can contribute to the stability and functional persistence of microbial consortia by reducing direct competition among consortium members. In pesticide-degrading communities, this partitioning may occur through metabolic division of labour, in which different microorganisms catalyse successive steps of pesticide transformation or utilize distinct degradation intermediates. For example, an atrazine-degrading consortium isolated from agricultural soil exhibited interspecies catabolism in which *Clavibacter michiganensis* ATZ1 initiated atrazine transformation, whereas *Pseudomonas* sp. CN1 utilized downstream metabolites such as N-ethylammelide and cyanuric acid; the consortium consequently degraded atrazine more rapidly than the initiating strain alone (de Souza et al. 1998). Experimental evolution of another atrazine-degrading consortium further demonstrated that labour sharing and metabolite-dependent interactions could promote coexistence among consortium members, thereby maintaining pesticide-degradation capacity (Billet et al. 2019). Such functional complementarity is also supported by soil-based studies, where inoculation with pesticide-degrading consortia substantially enhanced pesticide dissipation compared with uninoculated soil, including more than 20-fold enhancement of atrazine mineralization (Assaf and Turco 1994) and up to 94.3% degradation of chlorpyrifos in contaminated soil following bioaugmentation with a four-member bacterial consortium**.** Similarly, a three-member consortium of *Micrococcus aloeverae*, *Bacillus cereus*, and *Bacillus paramycoides* completely degraded malathion in soil within 15 days, outperforming the corresponding two-member combinations (Dar and Kaushik 2023). Together, these findings indicate that metabolic specialization and sequential utilization of pesticide-derived intermediates can facilitate coexistence and enhance the overall degradation capacity of microbial consortia.

### Self-regulation and adaptive responses

Collective stress response and self-regulation mechanisms are key elements that result in higher adaptability of microbial consortia to environmental conditions. The consortium members change the population during sub-optimal condition where the condition of pH variation, temperature, or high concentration of contaminants in water needs to be balanced and degradation capacity remains unaffected (Pegu et al. 2025). The ability to degrade acetamiprid was found in the acetamiprid-degrading consortium ACE-3 at a much wider pH range (6.0–8.0) and temperature range (20–42 °C) compared to individual isolates. This environmental flexibility is achieved through a population restructuring that makes those individuals that are better able to deal with stress more fit in the face of stress, and enriches the metabolic diversity of the population in the process. (Xu et al. 2020).

### Growth promotion through metabolite cross-feeding

The degradation intermediates could be used as substrates by the consortium members or they could be used as nutrients by the consortium members which create positive feedback loop and increase the overall productivity of the consortium. The metabolites such as amino acids, vitamins, cofactors, carbon compounds etc. are exchanged among the members of the community, this exchange is called as cross feeding relationships. In consortium MF0904, the growth of initial degraders produced organic acids that were used by downstream community members and the release of nitrogen from the intermediates of the amino acids helped the growth of members of the community that were nitrogen-limited (Pang et al. 2020).

### Extracellular enzyme cooperation

Microbial enzymes are the main pathway for pesticide degradation, causing most of the pesticide degrading activities in soil and water. They are controlling the transformation, detoxification, and degradation of molecules into less toxic/non-threatening molecules and they dictate the dynamic resistance and ecological risks (Silva-Andrade et al. 2025) (Satish et al. 2017). The contemporary genetics and omics tools have assisted us to observe the interaction of enzymes and pesticide at a molecular scale. With the help of the metagenomic and the screening studies, new enzymes were discovered, expanding the range of enzyme-based bioremediation (Ansar et al. 2026).

**Fig. 7:**
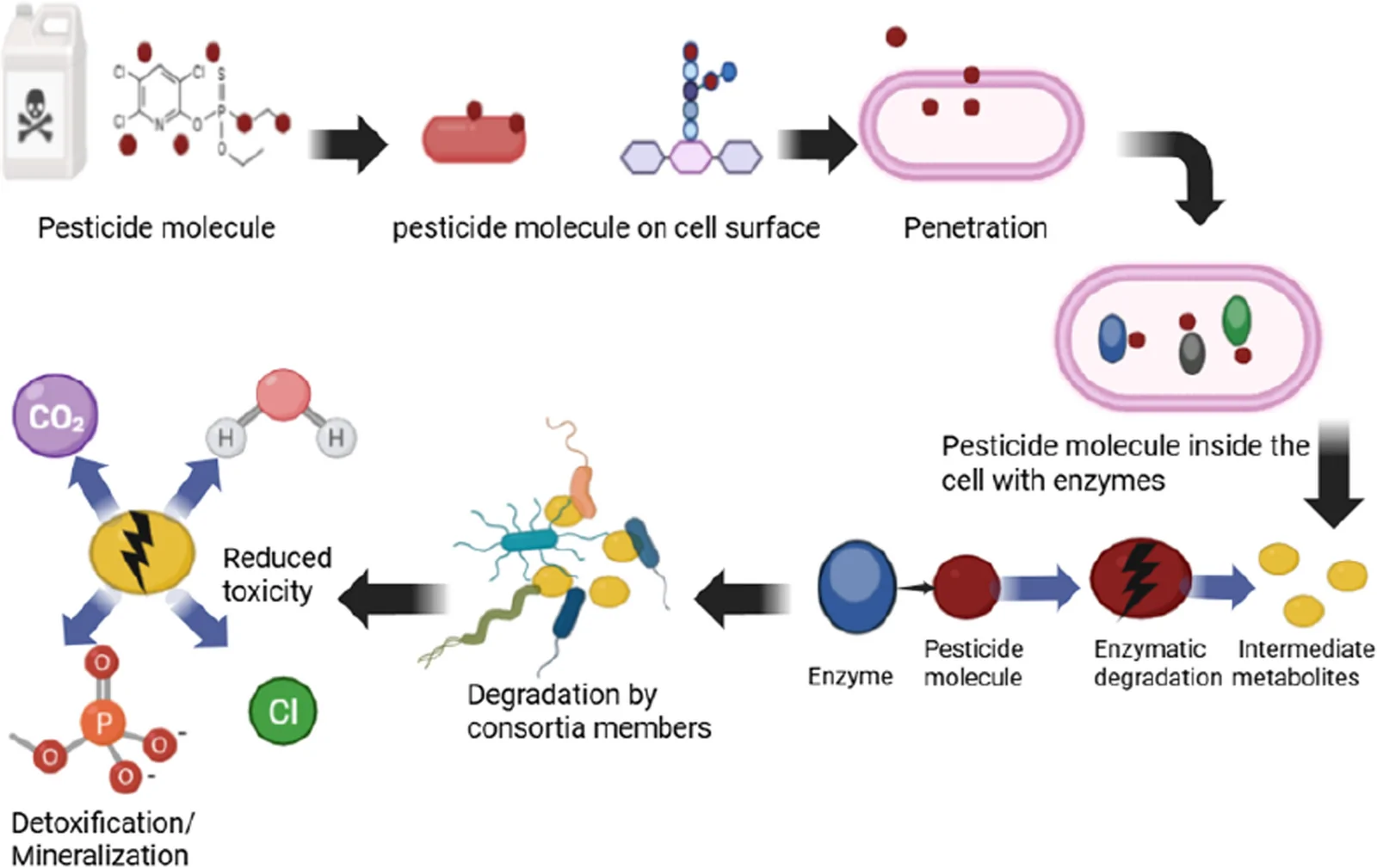
Pesticide biodegradation proceeds through a coordinated enzyme sequence—oxidative ring-opening by laccases/oxygenases, followed by hydrolysis (esterases, lipases, hydrolases), and reduction (nitro-reductases)—with each enzyme class typically supplied by a different consortium member

Recent studies focused on application of enzyme degraders of pesticides via enzyme immobilization, bio-augmentation, genetically engineered microbial systems to enhance bioremediation (Ansar et al. 2026). Several of the enzyme classes that are typically associated with them includes the hydrolases, oxygenase, peroxidases, reductases, lyases and glutathione-S-transferases. Hydrolases have a major role in mineralization of carbamates; an example of carbaryl hydrolase of the species in the genus *Arthrobacter* can hydrolyse carbamates. Often, oxidation is the first step in degradation and this is achieved by enzymes like laccases and oxygenase that break the aromatic rings and produce the reactive intermediates. After this, hydrolytic enzymes, which are esterases, lipases, cellulases assume the role and finally reduction proceeds by nitro reductases. These pesticides are modified in later stages by hydroxylation, alkylation, acylation and nitrosylation, and are finally completely detoxified (Jayasekara et al. 2025) (Fig. 7).

#### Hydrolases

The most well-studied group of enzymes in pesticide biodegradation is the hydrolases, which typically hydrolyse pesticide molecules via nucleophilic attack by water, either on an ester bond or a phosphoester bond or a carbamate bond. The initial step in organophosphate degradation involves the removal of an aromatic leaving group from the phosphoester bond by organophosphate hydrolases (OPH) and phosphotriesterases, thus greatly decreasing the acute neurotoxicity of the parent compounds. The most characterized is the *Brevundimonas diminuta* expressing OPH, which has a Km = 0.20 mM and kcat = 2100s-1 for the hydrolysis of chlorpyrifos, which is a high catalytic efficiency (Bose et al. 2021). The carboxylesterases play a crucial role in the metabolism of pyrethroids by hydrolysing the central ester bond to release cyclopropane carboxylic acid and aromatic alcohol which are then metabolized by central metabolic pathways. Under the field-relevant conditions, the esterase activity of the *Bacillus* and *Pseudomonas* species were confirmed to be the functional essentials for the dissipation of chlorpyrifos and malathion, and they are among the most frequent genera found to be hydrolase-producing in soils contaminated with pesticides (Verma et al. 2014).

#### Oxidoreductase

Oxidoreductases are enzymes that can transform the structure of the persistent aromatic pesticides by hydroxylation, ring cleavage, and dehalogenation, which in turn increase the polarity of the pesticides and their susceptibility to further enzymatic action (Islam et al. 2026). Cytochrome P450 monooxygenases are a prominent group of enzymes in this class, which is responsible for oxidative hydroxylation and N-dealkylation reactions that are key initial steps in the degradation of triazine herbicides, neonicotinoids, and chloroacetanilides (Liu et al. 2021)*. Stenotrophomonas* sp. Bacterial monooxygenase transformation of chlorpyrifos and TCP is an example for oxidative transformation products were confirmed by LC–MS, indicating enzyme-mediated, not adsorptive, pesticide removal (Malla et al. 2023). Laccases, lignin peroxidases, and manganese peroxidases are non-specific radical-generating enzymes that can oxidize organochlorines and neonicotinoids that are not likely to be attacked by the bacterial enzymes, thus providing exceptional diversity of oxidoreductases by white-rot fungi. This oxidoreductase property by the fungi is exemplified by *Trametes versicolor,* which degrades lindane at 72–88% by laccase mediated non-selective oxidation in 25–35 days (Satish et al. 2017; Chakraborty et al. 2025).

#### Transferases

Transferases, including glutathione S-transferases (GSTs) play an important role in detoxification of pesticide molecules and/or their reactive metabolites, by catalysing the conjugation of the pesticide molecule or its reactive metabolites with endogenous glutathione, which renders it more water-soluble, less biologically active, and more readily metabolized. GST-mediated conjugation is particularly significant for the reduction of bioavailability and toxicity of herbicide metabolites in polluted soil environments, where the presence of intermediate metabolites in free form, instead, would cause a prolonged ecotoxicological stress for non-target organisms (Pavlidi et al. 2018). *Sphingomonas* species are known to be important producers of GST in pesticide degrading consortia that provide the conjugation activity, along with the hydrolytic and oxidative activities contributed by other members of the community. Microbial consortia, in which multiple members of the community carry out the transferase activity, allows for continuous detoxification throughput even at high pesticide loading, whereas single-strain systems cannot sustain this throughput (Ansar et al. 2026). These enzyme classes rarely act in isolation within a consortium: extracellular hydrolases produced by one member often generate the substrate that a second member’s intracellular oxidoreductase or transferase then processes further.

## Major groups of pesticides and their degradation pathways

### Organophosphate pesticide degradation

Organophosphate Pesticides are generally more prone to biodegradation as compared to organochlorines since the former have labile phosphor-ester bonds in their chemical structure. A moderate-to-high soil adsorption coefficient (Koc = 1,000–6,070) and soil half-lives of 10–120 days nonetheless allow organophosphates to persist beyond a single growing season (Kumar et al. 2018). The organophosphate hydrolases or phosphotriesters are primarily responsible for catalysing hydrolysis of the phospho-ester bond, which is the initial step and usually the rate limiting step in the degradation of OP. The product of this reaction is aromatic alcohols, including 3,5-trichloropyridinol (TCP), and dialkyl phosphate derivates, which leads to significant decrease in neurotoxicity (Malla et al. 2023). After that, oxidative desulfuration reactions are catalysed by monooxygenases and oxidize the phosphothioate compounds to Oxon analogues. Oxon’s may temporarily become more toxic, but become quickly subjected to further enzyme transformation. The chronic TCP metabolites (dechlorinated, hydroxylated, have an aromatic ring cleavage) are catalysed by dehalogenase and oxygenase. At the same time, phosphorothioate side chains dealkylate and hydrolyse to produce inorganic phosphate in the end. The intermediates formed are directed to central metabolites, leading to complete mineralization of the intermediates to CO_2_, H_2_O, and inorganic ions. They can coordinate their enzyme processes well, thus efficiently and biodegrade the organophosphate pesticides into the environment. *Brevundimonas diminuta* OPH has Km = 0.20 mM and kcat = 2100s−1 which makes it highly catalytic (Bose et al. 2021). The genes mpd, encode the enzyme methyl parathion hydrolase (MPH) which has complementary substrate preferences showing higher activity towards methyl substituted organophosphates. (Kumar et al. 2018). MPH is a member of the Metallo-β-lactamase superfamily and has a different catalytic mechanism of action which is the nucleophilic attack on the phosphorus center with the participation of zinc-activated water (Efremenko and Sergeeva 2001). Despite well-characterised OPH/MPH-mediated hydrolysis of the parent compound, downstream mineralisation of TCP remains the rate-limiting and least field-validated step in organophosphate consortium degradation.

#### OP degradation pathway

##### Primary hydrolysis mechanisms

**Fig. 8:**
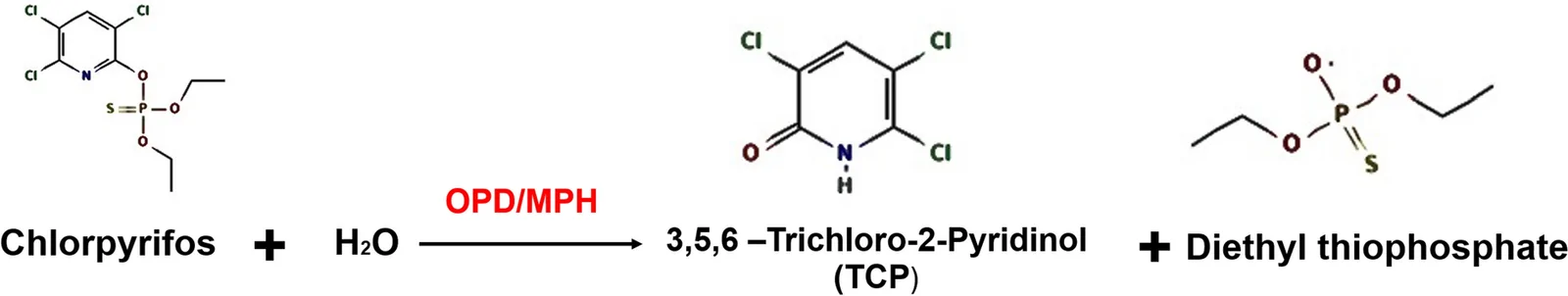
OPH/MPH-catalysed hydrolysis cleaves the phosphoester bond of chlorpyrifos, yielding 3,5,6-trichloro-2-pyridinol (TCP) and diethyl thiophosphate as the primary hydrolysis products

Chlorpyrifos degradation proceeds through two mechanistically distinct and sequential stages that should not be conflated: (i) primary hydrolysis, in which organophosphate hydrolase (OPH)/phosphotriesterase cleaves the phosphoester bond of the parent chlorpyrifos molecule to yield 3,5,6-trichloro-2-pyridinol (TCP) and diethyl thiophosphate (DETP), and (ii) further TCP degradation, a separate downstream sequence of reductive dechlorination, ring hydroxylation, and ring cleavage that converts the still-toxic TCP intermediate to non-aromatic, mineralizable products (Kumar et al. 2018; Arora and Bae 2014). Microbial degradation of organophosphates starts with hydrolysis of phosphoester bonds, which forms dialkyl phosphate and/or dialkyl thiophosphate intermediates, and a leaving group, usually an aromatic or a heterocyclic alcohol (Mulashkina et al. 2024). This primary hydrolysis is catalysed by phosphotriesterases, enzymes of various enzyme families and different structural and mechanistic properties (Fig. 8).

##### Downstream TCP degradation

**Fig. 9:**
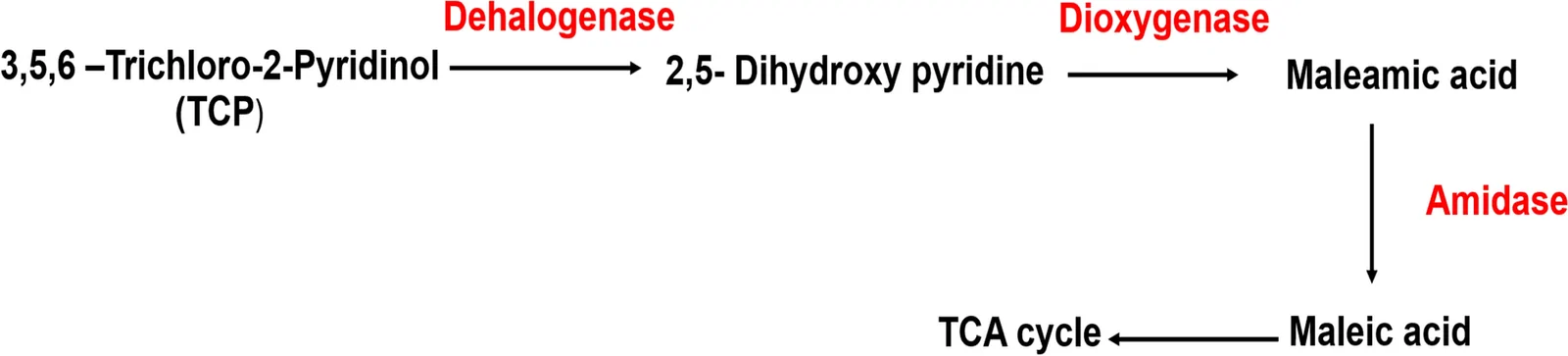
*Cupriavidus* sp. DT-1 dechlorinates TCP to 3,6-dihydroxypyridine−2,5-dione, which undergoes pyridine ring-opening to maleamic acid, feeding directly into the TCA cycle

Primary hydrolysis products need to be mineralized further. TCP is a moderately toxic intermediate, which needs to be ring-opened to detoxify it, which is produced when chlorpyrifos is metabolized (Kiran et al. 2025). The degradation of TCP involves reductive dechlorination and ring hydroxylation and cleavage. *Cupriavidus* sp. DT-1 catalyses the dechlorination of TCP to 3,6-dihydroxypyridine−2,5-dione, a compound which undergoes ring opening of the pyridine by a novel pathway yielding maleamic acid, a known intermediate of the tricarboxylic acid (TCA) cycle (Arora and Bae 2014). The steps that follow are the key elements of the pathway (Fig. 9).

The consortium method is important for the degradation of chlorpyrifos to phosphate and TCP, which requires organisms with efficient TCP degradation activity to also contain phosphotriesterase activity, and/or organisms with efficient phosphotriesterase activity to also contain TCP degradation activity. The consortium C3 (*Pseudomonas species, Bacillus* species, and *Stenotrophomonas* species) was able to degrade chlorpyrifos to 94% in 14 days while simultaneously *Pseudomonas* species contributed OPH activity and the *Bacillus* species and *Stenotrophomonas* species processed TCP intermediates (Malla et al. 2023).

##### Di alkyl phosphate metabolism

When chlorpyrifos undergoes hydrolysis, the diethyl thiophosphate group formed must be broken down by the action of phosphatase. In consortia, these reactions are catalysed by alkaline phosphatases, phosphodiesterases, the ethanol and sulphate released at this stage are assimilated into central carbon and sulphur metabolism rather than further transformed by dedicated pesticide-degrading enzymes, marking the functional endpoint of the pathway (Ramírez et al. 2023).

### Organochlorine pesticides

Some of the most persistent xenobiotic contaminants are organochlorine pesticides (OCP) like DDT, lindane, aldrin, dieldrin and endosulfan. Organochlorines typically show log Kow > 5.0 and environmental half-lives of years to decades, resisting all but the most specialised enzymatic attack (Li et al. 2021; Mansouri et al. 2017). High hydrophobicity and chemical stability are due to their high chlorination and hence they are also resistant to spontaneous hydrolysis, photolytic degradation, and microbial restructuring in ambient conditions. However, it has been empirically proven that it is thermodynamically feasible to enzymatic biodegrade OCPs even though the process is kinetically restricted in both aerobic and anaerobic environments (Li et al. 2021). The chief pathway of degradation is the hydrolysis of carbon chlorine (C–Cl) bonds; these are catalysed by other enzymes. Flavin dependent dehydrochlorinase. Stereoselective dehydrochlorination of the chloride ions by removing adjacent hydrogen Eg: LinA of the *Sphingobium*, which leads to the production of less-chlorinated diene intermediates. At the same time, nucleophilic substitution reactions can be catalysed by hydrolytic dehalogenases such as Lin B, where the aspartate residue in the active site reacts with the chlorinated carbon atom to form a transient acyl-enzyme complex, which is subsequently hydrolysed to produce the dechlorinated alcohol or alkene product and the free enzyme is recovered (Pavlidi et al. 2018; Rohilla et al. 2025).

The monooxygenases introduce hydroxyl and/or epoxide functional groups into the partially oxidized molecules. These groups are more polar and thus more susceptible to further enzymatic action. These are then acted on Fe (II)- dependent dioxygenases which oxidize aromatic rings at adjacent hydroxylated sites. These are then cleaved to give acetyl-CoA and fumarate which are used to supply central metabolic intermediates to the tricarboxylic acid cycle for complete mineralization (Khan et al. 2023; Chia et al. 2024). Fungal system is also important in the degradation of OCP. The results of (Satish et al. 2017) showed that the degradation efficiencies of lindane were 72–88% in fungal culture containing high lacticase activity for a span of 25–35 days. White rot fungi impact on the OCP transformation by non-specific oxidation, which is mediated by radicals under the catalysis of laccase and manganese peroxidases. The LC–MS analyses have determined chlorobenzene in the enzymatic transformation. However, it is found that degradation rates are much higher in biological active systems than in abiotic controls, based on the experiments of soil microcosms performed (Li et al. 2021).

#### Horizontal gene transfer

Importation of genes from a different organism by different mechanisms, known as horizontal gene transfer (HGT), plays a critical role in the evolution of microbial communities to new substrates to which pesticides are applied. The genes of pesticide catabolism are frequently plasmid-located, which makes it possible to spread rapidly within communities. The opd (organophosphorus-degrading) gene cluster, that encodes organophosphate hydrolase (OPH), was first found on a large plasmid in *Flavobacterium* ATCC 27551, and lab conjugation tests showed that the gene cluster could be transferred to *Escherichia coli, Pseudomonas putida* and soil strains with frequencies of the opd gene cluster encoding OPH, lab conjugation with 10^−3^ to 10^−4^ per donor cell per generation-rates high enough to explain the prevalence of OPH all over the world in contaminated soils (Xu et al. 2021). Recent metagenomic (2020–2025) studies on soils from agricultural farm fields have found that the CRISPR-Cas systems of indigenous microbes mediate adaptive changes perhaps through the control of mobile genetic elements in response to xenobiotics. The atzABC atrazine fragmentation cluster is located on the chromosome in the original *Pseudomonas* sp. ADP, it has now transferred to plasmid-encoded broad-host-range components in isolates at geographically remote locations- pure indications of recent HGT. Researchers have taken advantage of this natural form of genetic movement in bioaugmentation by placing catabolic plasmids of donor strains into an existing community (Chettri et al. 2024).

The degradation genes can be transferred from one member of the consortium to another via a process known as horizontal gene transfer, such as by conjugation, transformation, and transduction, which speeds up metabolic adaptation. Degradation genes are often linked to Mobile genetic elements (MGEs), such as plasmids, transposons and integrative conjugative elements that facilitate interspecies transfer. The cehA gene that encodes carbofuran hydrolase has been found on plasmids in several species of *Sphingomonas* and *Rhizobium*, indicating recent horizontal acquisition. Likewise, genes encoding the enzymes organophosphate hydrolase (opd and mpd) are linked to transposable elements that allow rapid spread between soil bacteria that are contaminated with organophosphate. High densities of cells and close cellular contact as seen in cooperative degradation creates ideal conditions for HGT in consortia. This genetic exchange ability can allow consortia to combine partial activities from individual members to form complete degradation pathways that the individual members do not possess.

Horizontal transfer of catabolic genes helps explain rapid field adaptation, but the recalcitrance of highly chlorinated ring structures means complete mineralisation—rather than partial dechlorination is still rarely demonstrated outside laboratory conditions.

### Carbamate pesticide degradation

#### Carbamate ester hydrolysis

**Fig. 10:**
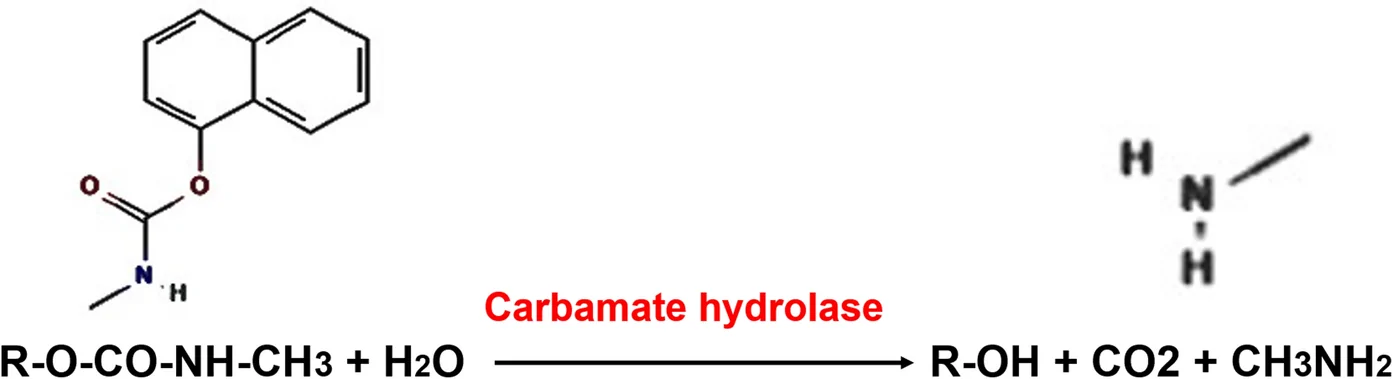
Carbamate hydrolase cleaves the –O–CO–NH– ester bond of carbaryl by nucleophilic attack on the carbonyl carbon, releasing 1-naphthol, CO2, and methylamine

The microbial attack is mainly directed towards the characteristic carbamate ester bond (–O–CO–NH–). This is cleaved by carbamate hydrolases by nucleophilic attack on the carbonyl carbon to give the aromatic alcohol or oxime, carbon dioxide, and methylamine (Fig. 10).

The enzyme, Carbaryl hydrolase (CaH) isolated from *Pseudomonas* sp. C5pp is an amidase with a signature of carbamates-cleaving enzymes, well characterised. For N-methyl carbamates, the enzyme is very specific and the enzyme kinetic parameters are Km = 125 μM and Vmax = 67.9 μmol/min/mg for carbaryl (Malhotra et al. 2021).

Carbofuran hydrolase (CfdJ) of *Novosphingobium* sp. Distinct substrate specificity was observed in KN65.2 with greater activity towards carbofuran and other benzofuranyl carbamates. The structural analysis showed that the geometry of the active site is responsible for the substrate selectivity; active site geometry is more favourable for the bicyclic benzofuran structure of carbofuran than the naphthalene ring of carbaryl (Nguyen et al. 2014).

#### Aromatic alcohol metabolism

**Fig. 11:**
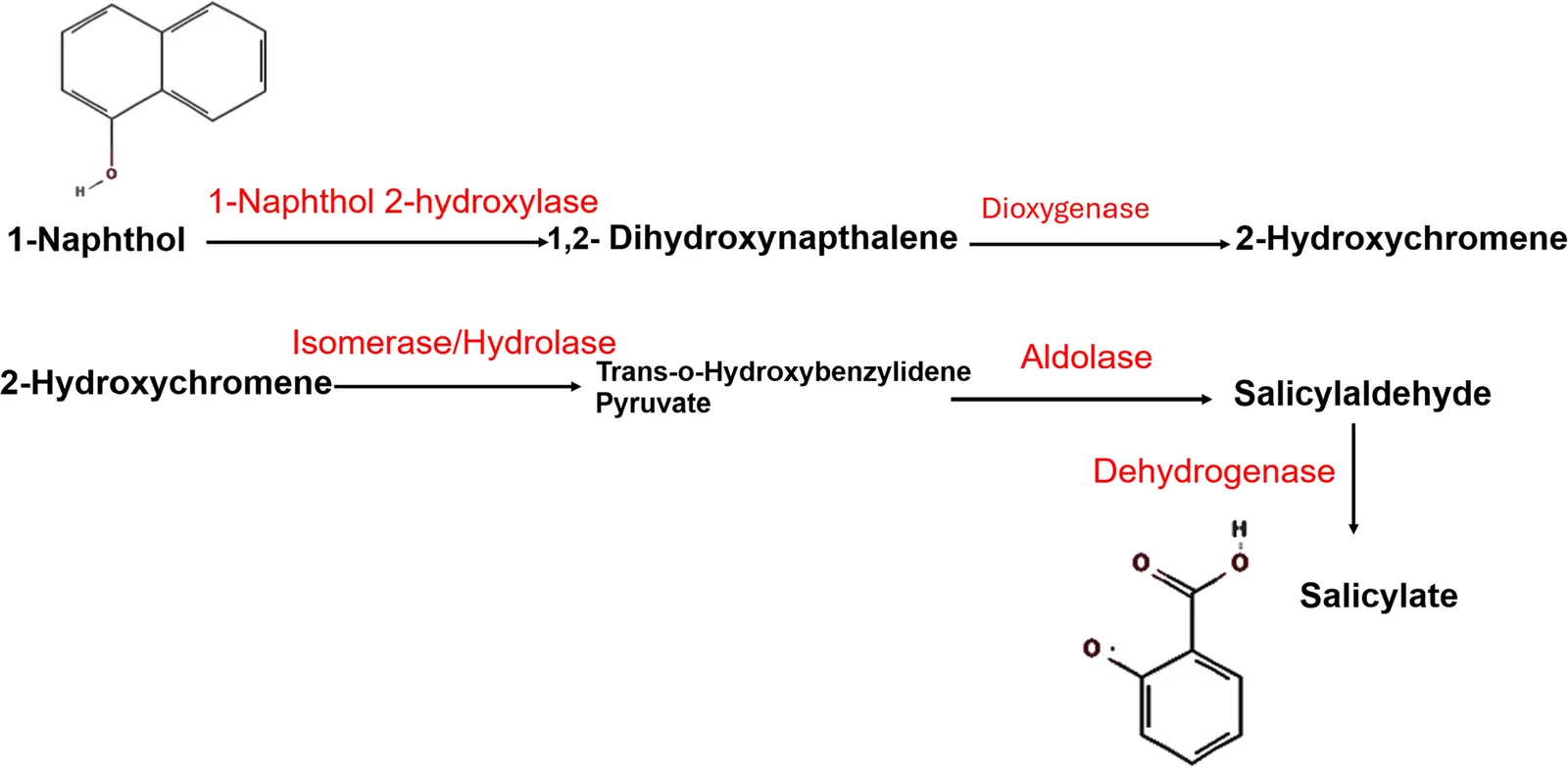
Naphthol from carbaryl hydrolysis is metabolised via two branches—the gentisate pathway (salicylate 5-hydroxylase) and the catechol pathway (salicylate 1-hydroxylase)—both converging on TCA-cycle intermediates

The carbaryl hydrolysis of 1-Naphthol takes place via hydroxylation to 1,2-dihydroxy naphthalene and subsequent ring cleavage to salicylate** (**Swetha et al. 2007). The pathway involves (Fig. 11).

Carbaryl hydrolase (CaH) from *Pseudomonas* sp. C5pp is a well characterized carbamate cleaving enzyme of the amidase signature family. The enzyme Salicylate metabolism then has two major branches that depend upon the organism that will break down the salicylic acid. The pathway is mediated by the enzyme salicylate 5-hydroxylase (S5H) that converts salicylate to gentisate which is then extra diol cleaved to maleylpyruvate. The catechol pathway is followed through the action of salicylate 1-hydroxylase (S1H) to yield catechol that can be metabolised by intra diol or extra diol ring cleavage pathways to yield intermediates of the TCA cycle **(**Rogers et al. 2019). The consortium BP16 of *Pseudomonas, Burkholderia and Rhodococcus* species achieved a mineralization rate of 95% in 96 h and were able to mineralize 100% of carbaryl in 96 h, whereas the most effective single strain mineralized 60% in 96 h because the pathway for 1-naphthol hydroxylase was distributed among the three species (Mohapatra and Phale 2021). Hydrolysis of carbofuran to carbofuran-7-phenol is followed by hydroxylation to the catechol 2,3-dihydro-2,3-dihydroxy-2-methylbenzofuran and ring cleavage reactions to yield the methylated catechol derivatives that are degraded in normal aromatic metabolism. (Mishra et al. 2021).

#### Methylamine assimilation

**Fig. 12:**
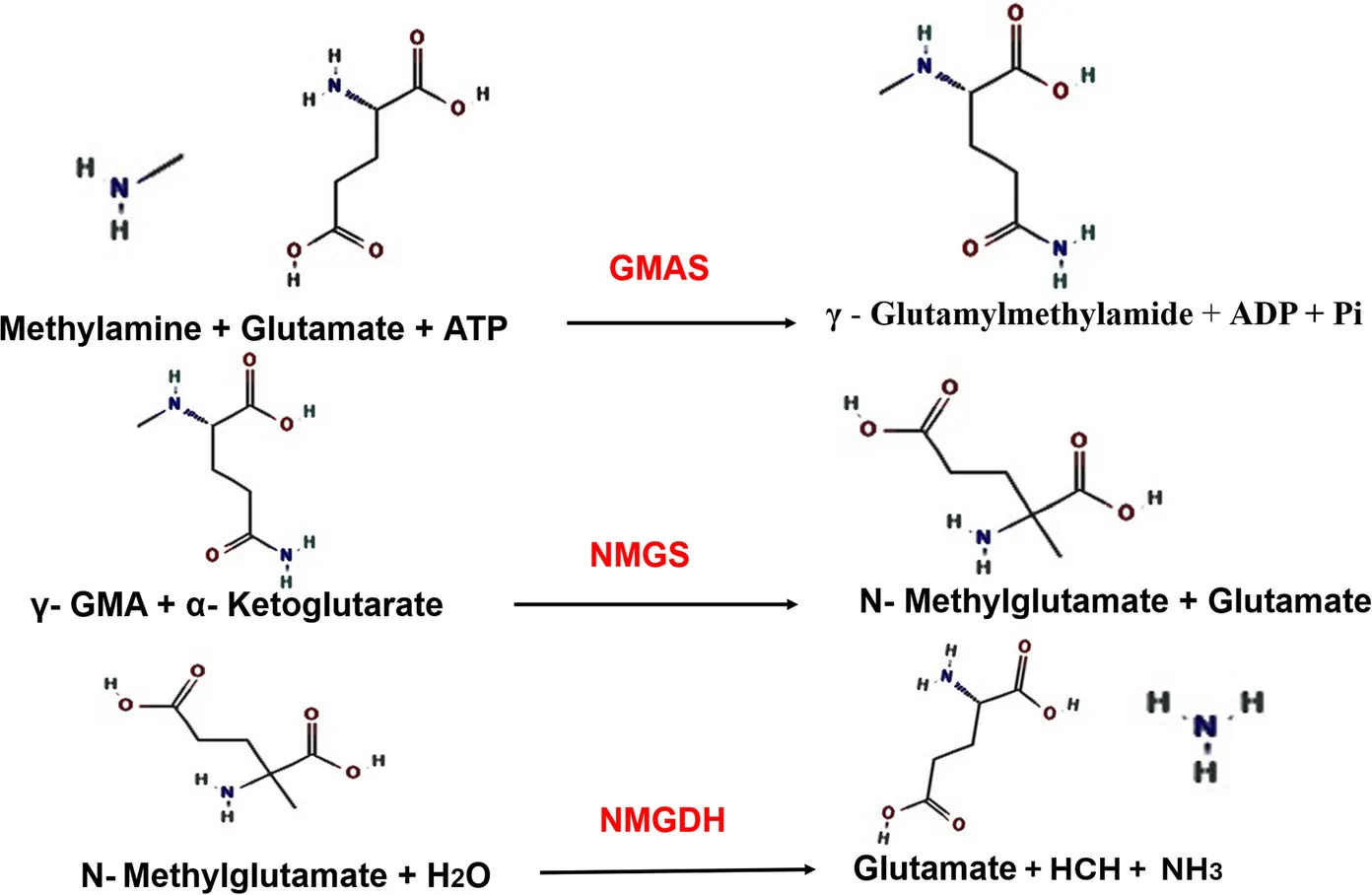
Methylamine released from carbamate hydrolysis is assimilated via NMGDH into central nitrogen metabolism, closing the carbamate mineralization pathway

Methylamine which is produced during the hydrolysis of the carbamates is a large nitrogen source available for assimilation in various ways (Kamini et al. 2018). The glutamate dependent pathway involves the condensation of methylamine with glutamate by γ-glutamylmethylamide synthetase (GMAS) to form γ-glutamylmethylamide (GMA), which is then converted back to glutamate and formaldehyde and ammonia by N-methylglutamate synthase (NMGS) and N-methylglutamate dehydrogenase (NMGDH) (Fig. 12).

Carbamate hydrolase activity is well documented at the single-strain level, but consortium-level studies confirming which member handles the resulting 1-naphthol or related phenolic intermediates remain comparatively scarce**.**

### Pyrethroid pesticide degradation

**Fig. 13:**
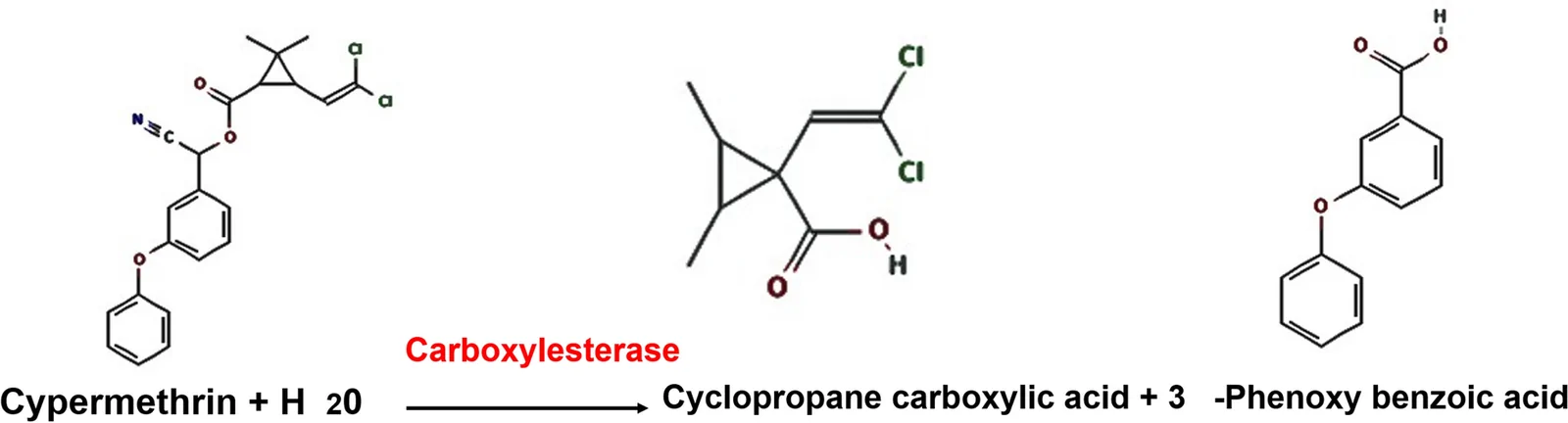
Carboxylesterases hydrolyse the central ester bond of cypermethrin, releasing 3-phenoxy benzoic acid (3-PBA) and dichlorovinyl-dimethyl cyclopropane carboxylic acid as the primary cleavage products

Pyrethroid mineralization requires two mechanistically separate stages that differ in the enzymes involved and in which consortium members typically perform them: Stage 1 (ester hydrolysis) cleaves the central ester bond between the acid and alcohol moieties of the parent pyrethroid, catalyzed by carboxylesterase-family pyrethroid hydrolases (Zhang et al. 2020), yielding an aromatic alcohol/aldehyde fragment (e.g., 3-phenoxy benzaldehyde) and an acid fragment (e.g., DCVA for cypermethrin). Stage 2 (aromatic ring cleavage of 3-PBA) acts on 3-phenoxybenzoic acid, the common and comparatively recalcitrant convergence intermediate of most pyrethroids, via dioxygenase-mediated ring hydroxylation and ether-bond cleavage to protocatechuate (Huang et al. 2018). These two stages are frequently performed by different consortium members, since many pyrethroid-hydrolyzing organisms lack the distinct dioxygenase system needed for 3-PBA ring cleavage (Fig. 13) (Tang et al. 2019).

#### Ester hydrolysis

Cypermethrin (α-cyano-3-phenoxy benzyl 3-(2,2-di chlorovinyl)− 2,2-dimethyl cyclopropane carboxylate) breaks down to 3-phenoxy benzaldehyde (or the corresponding alcohol) and 3-(2,2-di chlorovinyl)− 2,2-dimethyl cyclopropane carboxylic acid (DCVA).

Est3385 from *Rhodopseudomonas palustris* PSB-S is a new pyrethroid esterase that has a wide substrate spectrum that includes cypermethrin, deltamethrin and permethrin. The best catalytic activity of the enzyme occurs at pH 7.5 and temperature of 40 °C, and the catalytic efficiency of the enzyme for cypermethrin is expressed by the kinetic parameters, such as the catalytic efficiency (kcat/Km = 2.4 × 10^5^ M⁻^1^ s⁻^1^) (Zhan et al. 2020).

#### Aromatic ring cleavage of 3-PBA

**Fig. 14:**
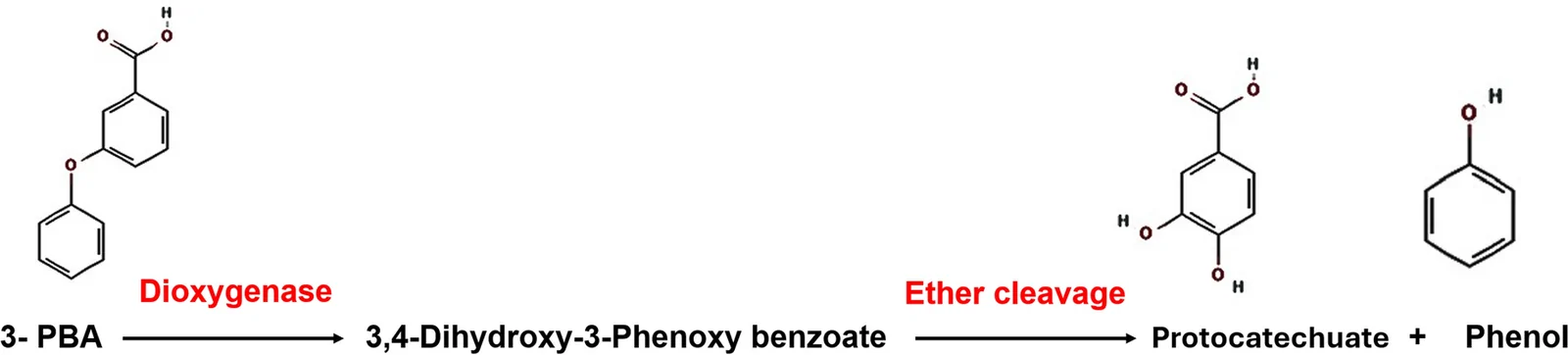
Ether-bond cleavage converts 3-phenoxy benzoic acid to protocatechuate, which then enters ring-cleavage metabolism via the ortho- or meta-pathway

3-Phenoxy benzoic acid (3-PBA) is a common intermediate of several pyrethroids that must be removed by ring cleavage to be fully degraded (Tang et al. 2019) (Fig. 14). The consortium YC-L1 consisting of *Bacillus, Stenotrophomonas and Sphingomonas* species degraded 3-PBA by ring hydroxylation by dioxygenase and then by cleavage of the ether bond:

Protocatechuate is then able to enter established aromatic degradation pathways through ortho-ring or meta-ring cleavage. The consortium approach becomes very useful as 3-PBA is more recalcitrant than parent pyrethroids, and many organisms degrading pyrethroids do not have the capability for 3-PBA degradation (Huang et al. 2018).

#### Metabolism of cyclopropane carboxylic acid

Cypermethrin is dechlorinated in two successive steps to form the DCVA moiety and then cyclopropane ring is broken. *Micrococcus* sp. strain CPN 1 initiates DCVA ring opening by reductive dechlorination while consortium members such as *Pseudomonas* species complete ring opening and carbon channelling to central metabolism. (Huang et al. 2018).

Ester hydrolysis to 3-PBA is consistently demonstrated, but 3-PBA itself is a persistent, moderately toxic intermediate whose further ring-cleavage and mineralisation by consortium members is the least-resolved step in the pathway.

### Neonicotinoid degradation pathways

#### Acetamiprid degradation

**Fig. 15:**
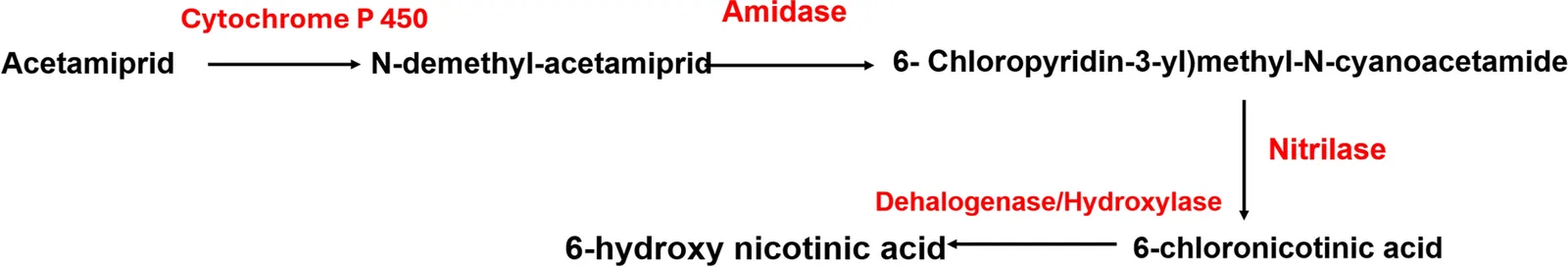
Consortium ACE−3 transforms acetamiprid through sequential oxidative and hydrolytic steps identified by LC–MS, active across a broad pH (6–8) and temperature (20–42 °C) range

These transformations of acetamiprid to various intermediate products by consortium ACE−3 are multiple transformations that can be identified using LC–MS analysis (Xu et al. 2020). Main pathways of degradation are (Fig. 15):

### Imidacloprid and clothianidin degradation

Imidacloprid is the most widely used neonicotinoid insecticide, and has high water solubility (610 mg/L), is systemic in plants, and is known to be toxic to non-target pollinators, making it a challenging insecticide to remediate. When imidacloprid is degraded by microbial consortia, the first step in the degradation is via oxidative and hydrolytic pathways with the nitroguanidine fragment being the first point of enzymatic action. The major degradation pathway is the hydroxylation of the imidazolidine ring to form 5-hydroxy-imidacloprid as the major initial metabolite followed by hydrolytic ring opening to form imidacloprid-urea. The intermediates formed by further degradation are desnitro-imidacloprid and olefin-imidacloprid, both of which are toxic, and result in the generation of 6-chloronicotinic acid which is toxic but requires specific ring-cleaving organisms for complete mineralization (Ji et al. 2025).

It is especially important that the consortium degradation of imidacloprid consists of sequential transformations, because most of the individual bacterial isolates can degrade only one or two of the steps. *Pseudomonas, Sphingomonas and Stenotrophomonas* species are shown to be commonly found in consortia for the degradation of imidacloprid, and remove 80–92% of the pesticide within 7–14 days, whereas the individual strains remove 25–40% under the same conditions. The chlorinated pyridine intermediate 6-chloronicotinic acid is produced during degradation of both imidacloprid and acetamiprid, and is therefore a significant metabolic convergence point that needs to be detoxified by a consortium member with the ability to dehalogenate it (DPR 2024).

The microbial consortia degradation of clothianidin also involves triazoline ring modification and transformation of the guanidine group. Soil microcosm testing has shown that polluted soils contain high levels of mineralizing consortia that can degrade 100% of the clothianidin in 21–28 days under optimal soil and environmental conditions, where the composition of the microbial communities consistently identified *Aminobacter, Mesorhizobium* and *Variovorax* species as important functional degraders. The potential environmental issues linked to neonicotinoids, including parent compound persistence in soil, systemic distribution in pollen and nectar, highlights the need for the development of a strong consortium-based remediation approach to not only eliminate parent compounds, but to also remove biologically active transformation products (Matsuda et al. 2020). Consortium-level degradation of imidacloprid and clothianidin to desnitro-imidacloprid is documented, but data on further mineralisation of this metabolite, which retains measurable toxicity, remain limited.

### Herbicides degradation

Of all the triazine herbicides, atrazine, simazine and propazine are the most used in the world and have been detected at concentrations exceeding the maximum limits in surface and ground water which has stimulated microbial degradation pathway studies on triazines (Hussain et al. 2015). The resistance against enzymatic degradation by the symmetric triazine ring is also high and it takes the concerted action of several enzyme systems from various members of the consortium for complete mineralization.

The *Pseudomonas* sp. one of the few single organisms that can break down atrazine to some extent is ADP strain which contains the atz gene cluster that encodes the entire atrazine-to-cyanuric acid pathway, but subsequent ring-cleavage (to cyanurate) requires organisms containing the cyanuric acid amidohydrolase (trzD) gene (Wackett et al. 2002). Atrazine degradation is best understood as two enzymatically and genetically distinct modules. Module 1- upper-pathway hydrolysis (atzA, atzB, atzC genes) which sequentially dechlorinates and deaminates atrazine via hydroxy atrazine and N-isopropylammelide to cyanuric acid; this module is frequently carried entirely within a single organism (e.g., *Pseudomonas* sp. ADP) because the three atz genes are typically co-located and function well independently of ring-cleavage capacity (Sajjaphan et al. 2004). Module 2—downstream cyanuric acid ring cleavage and assimilation (atzD/trzD, atzE, atzF genes) which hydrolyses cyanuric acid to biuret, then to allophanate, and finally to CO2 and ammonia via allophanate hydrolase, releasing nitrogen that can be assimilated into microbial biomass; this ring-cleavage module is frequently absent from atrazine-hydrolysing strains and must be supplied by a different consortium member (Wackett et al. 2002; Sajjaphan et al. 2004). It is the genetic separation of these two modules across different organisms rather than any single strain’s incomplete gene complement that explains why complete atrazine mineralization in soil is a consortium-level, not a single-strain, phenomenon (Mandelbaum et al. 1995).

In the natural soil environment degradation of atrazine may be carried out by consortia in which the members of the consortium may contain complementary subsets of the gene complement for degradation of atz and trz, such as *Arthrobacter* sp.*, Nocardioides* sp., *and Pseudomonas* sp. (Sajjaphan et al. 2004).

A captivating example of synergism in consortium degradations in degradation of atrazine was provided by Mandelbaum et al. (1995) who demonstrated that three bacterial strains alone caused no complete atrazine mineralization, but that complete evolution of 14CO2 from ring labelled 14C-atrazine was achieved in 14 days when the three strains were members of a consortium. The study was continued on the molecular level and revealed that each strain contained a different complement of enzymes that carry out different enzymatic steps in the degradation pathway, the example of metabolic division of labour in a degradative consortium. The degradation of 2,4-Dichlorophenoxyacetic Acid (2,4-D) occurs in both the atmosphere and water.2,4-Dichlorophenoxyacetic Acid (2,4-D) degrades in both water and the atmosphere.

#### 2,4-Dichlorophenoxyacetic acid (2,4-D) and phenoxyacetic herbicide degradation

2,4-Dichlorophenoxyacetic acid (2,4-D) is an extensively used broadleaf herbicide, and one of the most thoroughly investigated xenobiotics for microbial bioremediation research. It degrades by breaking the phenoxy ether bond between the phenoxy and acetic acid groups, after which the chloride is removed and the aromatic ring is hydroxylated to form chlorocatechol intermediates which go into the modified ortho-cleavage pathway for complete mineralization. The key enzymes for degradation of 2,4-D are part of a gene cluster (tfd) and are among the most well characterized genes in environmental microbiology, and often carried on conjugative plasmids which allow their horizontal transmission across soil bacteria. (Iasakov 2023).

In the environment, 2,4-D degradation by consortiums is consistently superior to monocultures even though some strains like *Cupriavidus pinatubonensis* JMP134 have the entire tfd gene cluster and can grow on 2,4-D alone. Bacterial consortia containing *Burkholderia* species*, Sphingobium* species, and *Comamonas* species have shown that 2,4-D degradation can be completed within 10–15 days in contaminated agricultural soils through metabolic cooperation, in which the initial 2,4-D degradation taking place in the soil by *Burkholderia* species produces 2,4-DCP, which is further dechlorinated and ring-cleaved by the complementary members of the consortia (*Sphingobium* and *Comamonas*) (Góngora-Echeverría et al. 2020). Góngora-Echeverría et al. (2020) evaluated biobed microbial consortia that showed excellent performance in the degradation of 2,4-D as part of a multi-herbicide contamination scenario, highlighting the applicability of microbial consortia-based approaches to mixed-herbicide contamination profiles widely observed in real agricultural environments.

#### Glyphosate degradation

**Fig. 16:**
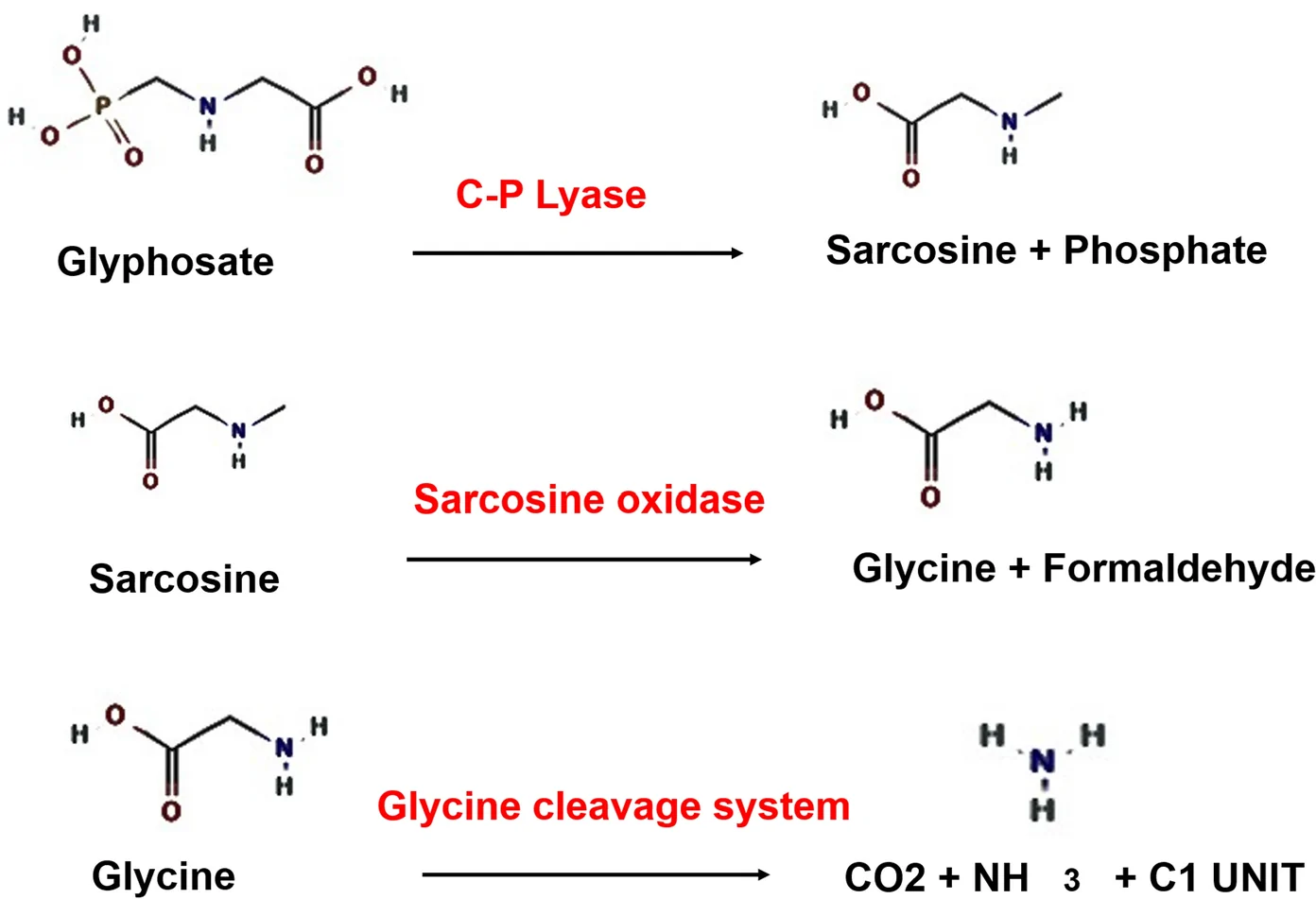
Field-trial consortia degraded glyphosate via the AMPA pathway to ammonia and inorganic phosphate, achieving an 85% reduction in field trial

The most widely used herbicide in the world is glyphosate (N-phosphonomethylglycine), leading to a high remediation need in the environment. Microbial degradation of glyphosate proceeds via two parallel, mutually exclusive enzymatic routes that consortia must reconcile. The C–P lyase (sarcosine) pathway, encoded by the phn operon, directly cleaves the carbon–phosphorus bond to yield sarcosine and inorganic phosphate; sarcosine is then oxidised via sarcosine dehydrogenase/oxidase to glycine, formaldehyde, and ultimately central metabolites (Souza et al. 2025). This route dominates under phosphate-limited conditions and is considered ecotoxicologically preferable, since it does not generate AMPA (Zhan et al. 2020). The glyphosate oxidoreductase (GOX) pathway instead cleaves the carbon–nitrogen bond, producing aminomethylphosphonic acid (AMPA) and glyoxylate; glyoxylate is assimilated into microbial biomass or the glyoxylate cycle, but AMPA itself requires a separate C-P lyase step (again via the phn operon) for further breakdown to inorganic phosphate and methylamine (Dar et al. 2026). AMPA is markedly more persistent and more frequently detected in soil and water than glyphosate itself, and its accumulation rather than parent-compound removal is now considered the more relevant endpoint for assessing true glyphosate detoxification (Aslam et al. 2023). The coordinated C-P bond cleavage, glycine oxidation and phosphate assimilation by the consortium YS622 was enough for complete glyphosate mineralization (Tang et al. 2021) and consortium designs that combine both GOX- and C-P-lyase-competent members are best placed to prevent AMPA accumulation regardless of which initial route a given soil community favours (*Comamonas odontotermitis* P2 has been shown to carry both pathways in parallel (Aslam et al. 2023). Dominant genera were identified as *Pseudomonas, Achromobacter and Comamonas*, during community analysis, and were determined to provide complementary enzyme activities. The C-P lyase pathway goes through the following steps (Fig. 16):

The consortium introduced the concept of contaminated soil bioremediation and the field trial showed an 85% reduction in glyphosate after 30 days of treatment.

Across the six pesticide classes reviewed here, the strength of mechanistic evidence is uneven. Organophosphate hydrolysis via OPH/MPH is the most experimentally validated pathway, with characterised genes (opd, mpd) and defined intermediates (TCP, DETP) supported by gene-level and enzyme-activity data. Pyrethroid ester hydrolysis to 3-PBA and glyphosate degradation via the C-P lyase and glyphosate oxidoreductase routes are similarly supported at the gene level. By contrast, evidence for downstream triazine cyanuric-acid metabolism, and for several carbamate and neonicotinoid consortium interactions, remains largely inferred from single-strain studies extrapolated to consortium behaviour rather than from consortium-level gene-expression or knockout data confirming which member performs which step. Field-validated evidence (as opposed to laboratory-only) is comparatively strongest for organophosphates and select organochlorines; pyrethroid, neonicotinoid, and triazine consortium degradation remain almost entirely laboratory- or microcosm-scale*.* The C-P lyase and glyphosate-oxidoreductase routes are both characterised at the gene level, but which pathway dominates under field soil conditions and how consistently AMPA is further mineralised rather than accumulated remains an open, poorly field-validated question*.*

## Molecular mechanisms of enzyme promiscuity and substrate recruitment

Enzyme promiscuity means that enzymes catalyse reactions on substrates other than those for which they are physiologically evolved which is playing a central role in the evolution of the pesticide degradation pathways. Promiscuous enzymes give it some initial activity against a novel structure of a pesticide, which leads to selection pressure for optimization of the pathway by mutation and horizontal gene transfer. In the hydrolase superfamily, a wide range of pesticide substrates are promiscuously hydrolysed. The enzyme carbaryl hydrolase (CH) is produced by the bacteria *Pseudomonas* sp. C5pp has been evolved to degrade carbaryl, but is active toward several N-methyl carbamate pesticides such as propoxur and methomyl. Organophosphate hydrolases (OPH) were initially discovered for their ability to break down nerve agents, but they have also been found to hydrolyse a wide variety of other organophosphates used in agriculture, such as chlorpyrifos, parathion and malathion (Gupta 2016). In consortia, enzyme promiscuity can facilitate adaptation to mixed pesticide contamination since the enzymes have some activity against multiple substrates, which can be useful for adapting rapidly to it. Following optimization is by selection of consortium members who exhibit increased activity towards predominant contaminants and diversity for flexibility of substrates (Copley et al. 2023).

These pathways of degradation are not unique and efficient in consortia; they can be significantly influenced by both the community structure along with environmental factors, and by the capacity to design and engineer degradation pathways in a more efficient manner.

## The factors which affect the efficiency of microbial consortia

### Environmental parameters

Consortiums performance is significantly influenced by pH, temperature, and pesticide concentration: Consortiums ACE-3 were relatively pH tolerant (3–8), and temperature tolerant (20–42 °C) with an optimum range (Xu et al. 2020). Consortia of fipronil and thiobencarb had a decrease in degradation rates at higher concentrations (25–800 µg/mL) of the pesticides. Exposure to this toxin has been shown to cause toxicity effects on enzyme activity, metabolism, and growth (Faridy et al. 2024; Ramírez et al. 2023).

### Nutritional and redox conditions

Availability of electron acceptors, carbon and nitrogen affects community composition and activity. The secondary substrates are necessary for co-metabolic degradation, and additional carbon and nitrogen is needed for the capacity of enzymatic degradation may decrease Microbial consortia with higher degradation are found in sediment, sewage sludge, and the fertile soils. Nutrient rich conditions and pre-adaptation are likely the reason for the efficiencies (Tyson et al. 2004).

### Community’s composition and compatibility

Functional vs auxiliary members: A subset of genera in L1 had predicted degradation genes, and auxiliary taxa modulated the process indirectly, both were essential but excess of taxa in the auxiliary made a difference for the worse performance (Li et al. 2022; Tyson et al. 2004).

### Competition vs cooperation

In engineering consortia, the attention is directed towards competition between microbes. The stability is determined by the relative contribution of two interactions: nutrients and cross-feeding (Xu et al. 2021; Cao et al. 2022a, b). Diversity and network structure changes of L1 community structure over time, but not the overall diversity was correlated with either loss or maintenance of the degradability, indicating that network organization is crucial than just species richness (Li et al. 2022).

## Advances

### Molecular profiling using omics technologies

The paradigm shift in pesticide bioremediation from conventional approaches to data-driven, technology-enabled methods has been enabled by advances in molecular biology, microbial ecology, synthetic biology, and artificial intelligence (Alavian and Khodabakhshi 2025a). Multi-omics approaches like metagenomics (DNA sequencing), transcriptomics (RNA profiling), proteomics, and metabolomics have transformed the study of pesticide-degrading microbial communities by enabling functional characterisation without the need for culturing (Hassan and Ganai 2023; Sengupta and Pal 2021). Metagenomics has identified chlorpyrifos-degradation genes in both cultivated and yet-to-be-cultivated soil microbes, including *Stenotrophomonas* sp. YC-1 (chlorpyrifos) and *B. pumilus* C2A1 (TCP), while proteomics has implicated hydrolytic and oxidoreductive enzymes such as esterases, dehalogenases, peroxidases, laccases in pesticide detoxification (Malla et al. 2023). Machine learning combined with multi-omics is increasingly used to predict degradation pathways and design synthetic consortia, and CRISPR-Cas-based synthetic biology now allows engineering of strains capable of degrading multiple pesticide classes (Dhakal et al. 2025). However, these workflows remain constrained by shallow sequencing depth in complex soil communities, difficulty linking specific genes to specific taxa without culturing, and the absence of standardised bioinformatic pipelines for cross-study comparison**.**

### CRISPR-based engineering

In the last several decades, biotechnology invented the latest tools of genome editing, such as CRISPR-CAS system, TALEN, Zinc -finger nucleases allow scientists to alter the sequence of the genome of the microorganisms. These technologies can be optimised to the development of the microbial consortia. It entails taking part in the designing adaptation in genes of microbes that are linked to the degradation of pesticides (Bhatt et al. 2021a, b; Zhang et al. 2023). The gene sequences of the enzyme involved in the degradation of the compounds like organophosphates, carbamates and other xenobiotics are integrated into the microbes in the laboratory which leads to the increase in the mineralization capacity of the microbes, that is, key enzymes (oph, mph etc.). This genetically engineered organism is performing better degradation efficiency and fast degradation rate. Furthermore, they can also withstand and live in toxic environment, and have high metabolic efficiency (Sun et al. 2015). Their capacity to upregulate their enzyme expression, their catabolic pathways and even show completely new biochemical pathways point to the potential of engineered microbes as next generation cleaning agents in a sustainable way to clean the environment (Alidoosti et al. 2024). Although gene expression systems based on the lactose operon are not used in microbial control in synthetic biology but are finding some applications such as microbial control using IPTG, it is also finding some limitations in practice as it is non-toxic to bacteria, and cost-effective alternative (Cao et al. 2022a, b).

First, containment is field release of any genetically engineered microorganism (GEM) requires demonstrated physical or genetic confinement; the only large-scale field trial of a catabolically engineered GEM to date (*Pseudomonas fluorescens* HK44, released at the Oak Ridge Reservation to degrade PAHs) showed that escape beyond containment lysimeters occurred only sporadically, chiefly under low humidity and low wind speed, illustrating that even successful containment is conditional on environmental factors rather than absolute (Ripp et al. 2000). Modern intrinsic-biocontainment approaches kill switches, toxin-antitoxin systems, synthetic auxotrophy, and CRISPR-based kill switches are increasingly used to reduce this risk for engineered strains intended for open release (Palladino et al. 2024). Second, horizontal gene transfer (HGT): engineered catabolic genes carried on conjugative plasmids can transfer to indigenous soil bacteria, which can be leveraged deliberately as “genetic bioaugmentation” (introducing a catabolic plasmid rather than a whole donor strain) shown to achieve complete breakdown of a model xenobiotic in soil microcosms within 8 days while imposing only low fitness cost on the recipient host but the same mechanism also means engineered traits cannot be fully recalled once released (Marquiequi 2025). Third, fitness cost: engineered strains carrying additional catabolic burden often show reduced competitiveness against indigenous soil microbiota, which is a major reason why laboratory degradation rates are rarely reproduced at field scale. Fourth, regulatory constraints: agencies such as the U.S. EPA and the European Food Safety Authority require survivability, genetic-stability, and ecological-impact data before permitting field deployment of any GEM, and current frameworks remain more developed for contained bioreactor applications (e.g., wastewater treatment) than for open-soil release of pesticide-degrading consortia, a key reason CRISPR-engineered consortia remain, for pesticide bioremediation specifically, at a laboratory or contained-pilot readiness level rather than field-validated status (Olawade et al. 2025).

### Designed consortiums and immobilization technologies

A microbial consortium is more efficient than a single-strain system. They have a functional complementarity and metabolic division of labour. Carefully designed combinations of bacteria such as *Pseudomonas, Bacillus, Achromobacter*, and *Rhodococcus* enable them to cooperate in the degradation of pesticides, such as chlorpyrifos, as well as reducing the amount of toxic intermediate pesticides, such as TCP (Kiran et al. 2025). Rhizoremediation also improves the degradation by activating the microbial activities induced by plant root exudates, which activates the enzyme(s) involved in pesticide degradation.

One of the big strides in consortium remediation is immobilization technologies. The ability of microbial consortiums to become matrix-bound, such as alginate, biochar or polymeric carriers, increases the survival of the microbes, resistance to adverse environmental conditions, and prolongs their soil system function. The immobilized consortia exhibit enhanced degradation ability, minimal microbial washout, and usability, which are identified as some of the major limitations of free-cell application in the field (Praveen and Nagalakshmi 2022). Immobilisation on carriers such as alginate beads, biochar, or activated carbon improves inoculum survival in laboratory trials, but carrier cost, scale-up manufacturing, and variable performance once carriers are exposed to field-level moisture and temperature fluctuation remain the main barriers to translating these gains beyond pot-scale trials.

### Artificial intelligence and computational modelling

Optimisation of microbial consortia using artificial intelligence (AI) and computational modelling is gaining in popularity, with current, demonstrated applications falling into five categories which were Prediction of enzyme–substrate interactions, using sequence or structure-based models to flag candidate pesticide-degrading enzymes ahead of wet-lab screening; Strain and taxon classification from sequencing data, accelerating functional annotation of metagenomic datasets; Modelling of community dynamics, using genome-scale metabolic models and flux-balance approaches to simulate cross-feeding and predict which species combinations are metabolically compatible; Rational design of consortia composition, ranking candidate species combinations by predicted degradation performance before laboratory testing; and Integration of multi-omics layers (genomics, transcriptomics, proteomics, metabolomics) into unified predictive models of community function (Loganathan 2026; Blessing and Olateru 2025). These capabilities remain constrained by limitation in training datasets in pesticide bioremediation are typically small and drawn from heterogeneous lab conditions, limiting model generalizability; field validation of AI-derived consortium designs is rare, with most reported successes confined to laboratory or simulated conditions; and most current models are prediction-oriented “black boxes” with limited interpretability, which constrains practitioner trust and regulatory acceptance,a gap that explainable-AI (XAI) approaches (e.g., SHAP-based interpretive frameworks) are only beginning to address in environmental applications generally, and have not yet been systematically applied to pesticide-degrading consortium design specifically (Aravind et al. 2026).

### Synthetic microbial consortia engineering

Synthetic microbial consortia are two or more deliberately combined, often genetically modified, species which represent an advanced form of microbial engineering, already used commercially in bioaugmentation and biocontrol via well-characterised bacteria-fungi combinations (Chaudhary et al. 2023; McCarty and Ledesma-Amaro 2019). A key unresolved priority is rational design of minimal functional consortia: existing consortia are often large (more than 10 species) with many functionally redundant members, and applying flux-balance analysis and constraint-based metabolic modelling to identify the minimal subset of organisms and genes needed for full mineralisation would substantially simplify inoculant production, regulatory approval, and quality control. This reductiont design philosophy, already established in synthetic biology for metabolic engineering, has not yet been systematically applied to pesticide-bioremediation consortium design (McCarty and Ledesma-Amaro 2019; Cao et al. 2022a, b) and remains constrained in practice by an assembly-stability problem in which consortia optimised under sterile conditions frequently lose their intended structure once competing with indigenous microbiota in non-sterile field soil.

### Hybrid physio-chemical-biological remediation approaches

Biological remediation alone is often insufficient where contamination is severe, tightly sorbed to the soil matrix, or aged beyond ready bioavailability. Hybrid physico-chemical-biological approaches addresses nanoscale zero-valent iron (nZVI) pretreatment reduces organochlorine pesticides to partially dechlorinated, more bioavailable products and favours dehalogenating microbial communities that support subsequent consortium-mediated mineralisation, while TiO2-based photocatalytic oxidation and solar-driven advanced oxidation break recalcitrant aromatic rings into less toxic, more biodegradable intermediates (Li et al. 2024a, b). Plant–microbe hybrid approaches mobilising rhizosphere consortia via root exudates have reduced half-lives of persistent organochlorines such as DDT and DDE beyond what either phytoremediation or bioaugmentation achieves alone (Sun et al. 2015; Mansouri et al. 2017), and *Bradyrhizobium-*legume associations have reduced sulfentrazone residues by ~ 65% in agricultural field settings (Lopes et al. 2022a, b). Support-matrix co-formulation (biochar, alginate, clay minerals) further improves inoculant survival and sequesters toxic intermediates (Praveen and Nagalakshmi 2022). Hybrid systems overall offer better remediation performance, resilience, and cost-effectiveness than single approaches. Though pretreatment cost and the scarcity of comparative cost–benefit data against biological-only remediation still confine most hybrid systems to pilot-scale demonstration.

## Field-scale and pilot-scale applications

Most work on consortium-based pesticide bioremediation has been carried out in the laboratory or in a small-scale microcosm setting, but there has been a recent increase in small scale field and pilot-scale studies to help provide the needed evidence for the translation of these systems. One of the more practically proven Consortium applications is bio-bed systems, which are engineered bio-mixtures of straw, soil and peat that are used to capture and degrade pesticide runoff from agricultural point sources. Previous studies conducted by Góngora-Echeverría et al. (2020) have proven the effectiveness of bio-bed-derived indigenous microbial consortia in soil-straw bio-mixture systems, with more than 90% degradation of atrazine, carbofuran and glyphosate, establishing a proof-of-concept for on-farm pesticide waste management.

The reduction of chlorpyrifos residues in agricultural soil has been reported to reach 70–85% within 45–60 days of bioaugmentation with defined multi-species consortia in chlorpyrifos-contaminated soils, whereas it was reported to reach 30–40% in the same soils after natural attenuation (Lopes et al. 2022a, b). Field experiments using rhizoremediation technologies based on PGPR consortia and leguminous crops have achieved up to 65% degradation of sulfentrazone and organochlorines after one crop cycle, with an additional benefit of enhanced soil microbial diversity and crop yields (Lopes et al. 2022a, b; Sun et al. 2015).

Translation from laboratory to field is constrained less by degradation capacity than by whether an introduced consortium can persist once released. Survival depends on the ability of introduced strains to remain metabolically active under fluctuating field conditions and to withstand competitive pressure from the resident soil microbiota inoculum performance is further limited in zones of high or unevenly distributed contaminant concentration i.e., under soil heterogeneity (Srivastava et al. 2026). Carrier and delivery formulation (biochar, alginate beads, clay- or biopolymer-based matrices) is now treated as part of inoculum design rather than an afterthought, since it directly affects establishment against the indigenous microbiota and buffers the inoculum against moisture fluctuation. Repeated pesticide exposure and pesticide aging alter both bioavailability and the selective pressure the consortium experiences, so degradation rates measured on freshly spiked soil routinely overstate performance on aged, weathered field residues (Faridy et al. 2024). Regulatory approval pathways for bioaugmentation products remain slow and jurisdiction-specific, and the absence of standardised methods for monitoring introduced strains in situ strain-specific qPCR or metabarcoding markers rather than end-point residue sampling alone and Farmer-level adoption further depends on factors beyond biological performance, including product cost relative to conventional pesticide disposal, ease of on-farm application, and visible, timely evidence of soil remediation, none of which are yet systematically reported alongside degradation-efficiency data in the literature reviewed here.

## Challenges

**Fig. 17:**
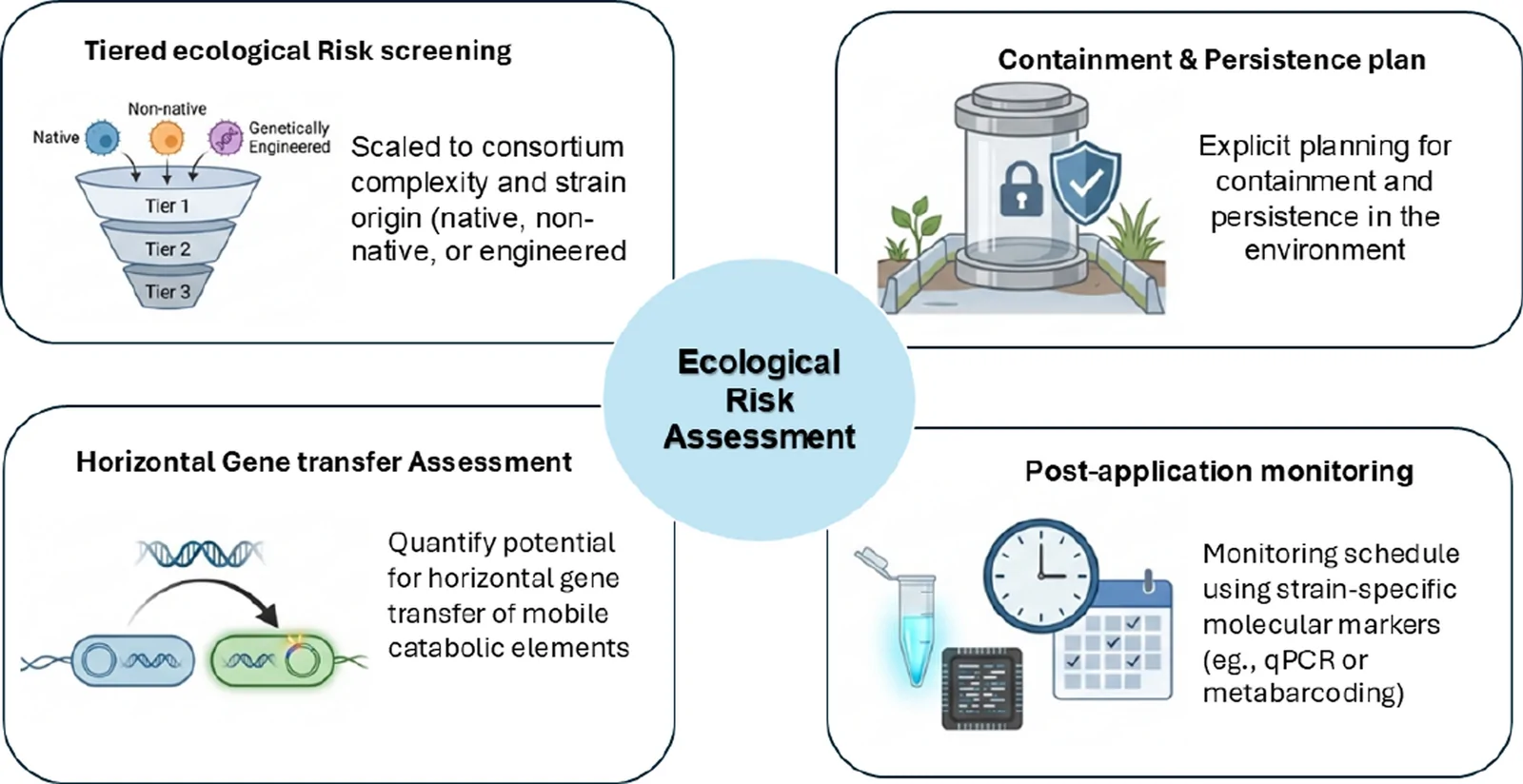
Recommended pre-release ecological risk assessment for consortium-based bioremediation products

Field deployment of pesticide-degrading consortia faces four interlinked challenges. First, functional stability: consortium performance depends on maintaining a defined community structure under fluctuating temperature, pH, nutrient availability, and inter-species competition, and destabilisation under environmental stress remains difficult to predict (Arslan et al. 2017; Dhakal et al. 2025). Second, interaction complexity: mutualism, competition, predation, and chemical signalling between community members jointly determine degradation efficiency, and this network complexity is one of the largest obstacles to controlling biodegradation outcomes at field or pilot scale (Wijayawardena and Naidu 2025; Rodriguez et al. 2025). Third, monitoring: real-time tracking of microbial dynamics is needed to optimise degradation, but current tools remain limited, optical density gives only coarse trends, PCR-based methods are slow single-time-point snapshots, and in situ sensors lack sensitivity to small compositional shifts (Wijayawardena and Naidu 2025; Raajaraam and Raman 2024). Fourth, regulation and intellectual property: registration of bioremediation products is costly and slow, requirements vary across authorities, and unclear ownership of synthetic consortia together with overlapping patents raise both commercialisation and freedom-to-operate barriers (Rodriguez et al. 2025; Ahmad et al. 2023). Because next-generation bioremediation products are increasingly multi-species consortia rather than single strains, ecological risk assessment must treat the consortium itself as the unit of evaluation. Horizontal gene transfer is a specific, measurable component of this risk: field microcosm studies in manure-amended soil show plasmid-borne genes from an introduced strain transferring into resident soil bacteria within days of application (Lerner et al. 2026) Biocontainment strategies such as auxotrophic dependency and genetic kill-switches reduce but do not eliminate this risk once cells leave controlled conditions. We recommend pre-release risk assessment for consortium-based products include the four elements set out in Fig. 17.

## Future perspectives

The future of microbial consortia for pesticide bioremediation lies not in a single technological fix but in the deliberate integration of previously separate fields. Three priorities stand out. First, interdisciplinary integration: combining systems biology, which maps metabolic networks and inter-species interactions, with synthetic biology, which enhances degradation efficiency and stability, will allow more holistic consortium design, aided by AI-based models that predict community behaviour and optimise pesticide-removal strategies (Dhaarani and Reddy 2025); machine learning and AI similarly stand to improve real-time monitoring, though small training datasets and limited model robustness remain open challenges (Alotaibi and Nassif 2024; Silva-Andrade et al. 2025). Second, enzyme engineering: advances in molecular biology and protein engineering are improving the efficacy and selectivity of degradative enzymes such as organophosphate hydrolases, and industry players including Novozymes and Ginkgo Bioworks are already applying gene and fermentation-optimisation approaches to address the cost and stability barriers that have historically limited enzyme-based remediation at commercial scale (Wang et al. 2022; Xu et al. 2021). Third, translation to practice: bridging the persistent gap between laboratory and field performance will require coordinated action across researchers, regulators, agricultural extension services, and industry, supported by pilot-scale demonstration projects across diverse soil, climatic, and contamination scenarios, with results reported openly regardless of outcome to build the evidence base regulators and farmers both need. Taken together, recent progress in synthetic biology, AI, and environmental monitoring makes precision, knowledge-driven pesticide bioremediation more attainable within the next decade than at any previous point.

### A rational framework for selection and deployment of pesticide specific consortia

**Fig. 18:**
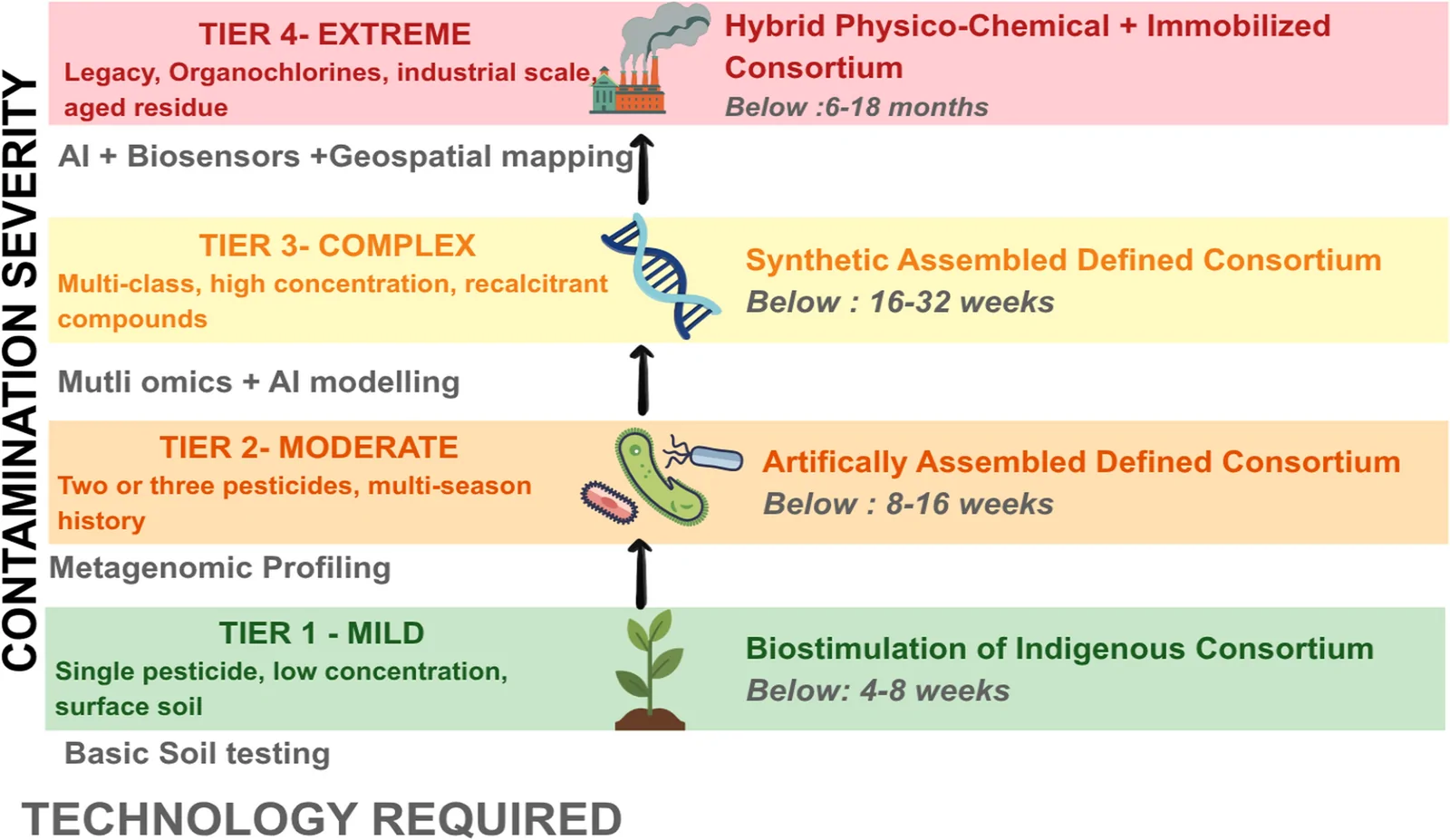
Tiered framework for microbial consortia selection based on contamination severity

Translating consortium-based bioremediation from bench to field requires matching consortium strategy to contamination severity. This review proposes a four-tier Consortium Selection Framework (Table 6; Fig. 18) spanning mild single-compound surface contamination (Tier 1: biostimulation of indigenous consortia, 4–8 weeks), moderate multi-class contamination (Tier 2: artificially assembled defined consortia guided by metagenomic gap analysis, 8–16 weeks), complex high-concentration contamination (Tier 3: synthetic or semi-synthetic engineered consortia with CRISPR-modified catabolic genes and multi-omics monitoring, 16–32 weeks), and extreme legacy contamination (Tier 4: hybrid physio-chemical pretreatment combined with engineered consortia). The framework ties each tier to a specific consortium type, assembly strategy, and technology support and is intended as a decision-support tool rather than a fixed prescription: tier assignment depends on site characterization rather than concentration alone, tiers are not mutually exclusive within a single heterogeneous site, and sites can move down tiers as remediation progresses (Lopes et al. 2022a, b; Dhakal et al. 2025).

**Table 6:** Different tier framework for selection and deployment of pesticide specific consortia

Parameter	Tier 1-mild	Tier 2 -moderate	Tier 3-complex	Tier 4-extreme
Contamination profile	Single pesticide class, low–moderate concentration, surface soil, recent history	Two to three pesticide classes, moderate concentration, mixed soil horizon, multi-season history	Multiple pesticide classes, high concentration, recalcitrant structures, decade-scale history	Legacy organochlorines, industrial-scale, aged residues, deep soil penetration
Representative pesticides	Chlorpyrifos, carbaryl, malathion (single application)	Chlorpyrifos + atrazine + carbofuran (combined)	Organophosphates + neonicotinoids + pyrethroids combined at high load	DDT, lindane, dieldrin, endosulfan at legacy contamination sites
Recommended consortium type	Naturally enriched indigenous consortium	Artificially assembled defined consortium	Synthetic or semi-synthetic engineered consortium	Immobilized hybrid consortium with physico-chemical pretreatment
Assembly method	Indigenous community enrichment through biostimulation	Laboratory selection and combination of specialist isolates with complementary catabolic genes	CRISPR-enhanced engineered strains assembled with ecological compatibility screening	nZVI or photocatalytic pretreatment followed by alginate or biochar-immobilized engineered consortium
Technology support required	Basic soil chemical analysis, pesticide residue monitoring, colony counting	Metagenomics profiling, degradation efficiency assays, community composition analysis	Multi-omics (metagenomics + transcriptomics + metabolomics), AI-driven composition modelling, real-time metabolite monitoring	Full AI-driven design, real-time field biosensors, geospatial contamination mapping, digital twin monitoring
Expected remediation timeline	4–8 weeks	8–16 weeks	16–32 weeks	6–18 months
Primary limitation	Insufficient for multi-class or high-concentration contamination	Requires careful strain compatibility screening; may underperform in highly toxic soils	Regulatory constraints on GMO field release; high technology cost	Very long timelines; high operational cost; site logistics complexity
Key supporting evidence	Góngora-Echeverría et al. (2020); Bhatt et al. (2021a, b); Dhakal et al. (2025)	Xu et al. (2020); Faridy et al. (2024); Shahid et al. (2023); Cao et al. (2022a, b)	Bhatt et al. (2021a, b); Alidoosti et al. (2024); McCarty & Ledesma-Amaro (2019); Srivastava et al. (2026); Trivedi et al. (2020)	Lopes et al. (2022a, b); Sun et al. (2015); Praveen & Nagalakshmi (2022); Kiran et al. (2025)

Prospective field validation of this framework across contamination types, soil types, and climates remains the key open task for establishing it as a standardized decision tool for researchers, practitioners, and regulators (Raajaraam and Raman 2024; Dhakal et al. 2025; Srivastava et al. 2026).

## Conclusion

The evidence presented in this review clearly demonstrates that microbial consortia is the most optimal biological system for 100% pesticide mineralization in contaminated environments. Functional consortia will overcome the metabolic incompleteness and toxic acidification problems of single-strain bioremediation; and with co-operative degradation and cross-feeding networks, metabolic division of labour is a defining feature. For all major pesticide classes studied, consortia degraded pesticides faster and degraded more varieties of chemicals and were more resilient to environmental stresses than monocultures. The enzymatic repertoire of the pesticide-degrading consortia, which includes hydrolases, oxidoreductases, transferases, and dehalogenases, demonstrates the catalytic diversity of microbial world and is responsible for multiple changes in complex xenobiotic structures to non-toxic end-products. However, the horizontal gene transfer and the presence of mobile genetic elements further promote adaptive evolution in community environments and allow for quick reactions to new contamination situations. A turning point towards rational design of bioremediation with systems-level approach has been achieved by combining multi-omics technologies, synthetic biology, computational modelling, and hybrid physio-chemical-biological approaches, thereby transitioning from empirical, strain-based remediation to rational, systems-level approach. Combined, these CRISPR-based engineering, immobilization strategies and AI-driven consortium optimization raise the predictability, scalability, and field applicability of consortium-based interventions. The use of plant–microbe partnerships for rhizoremediation also adds to the ‘toolbox’ for in situ pesticide removal. However, scaling up laboratory achievements to the field is a great challenge. The instability of microbial communities in response to changing environments, the complexity of the inter-species regulatory networks, the lack of proper real-time monitoring, and the regulatory and intellectual property framework surrounding engineered microorganisms all need further interdisciplinary research. Commercialization will rely on adaptive regulatory frameworks that can balance biosafety and innovation, and that are applied wisely. In the future, the potential for using systems biology, synthetic ecology, and AI-driven platforms for designing consortia with programmable degradation capacities, environmental responsiveness, and long-term ecological stability is transformative. Microbial consortia offer a scalable, environmentally compatible route to natural attenuation of pesticide-polluted ecosystems and represent one of the most promising directions for scaling bioremediation from the laboratory to the field.

## Data Availability

No datasets were generated or analysed during the current study.
